# Radiosensitization of Osteosarcoma Cells Using the PARP Inhibitor Olaparib Combined with X-rays or Carbon Ions

**DOI:** 10.7150/jca.90371

**Published:** 2024-01-01

**Authors:** Meng Dong, Hongtao Luo, Ruifeng Liu, Jinhua Zhang, Zhen Yang, Dandan Wang, Yuhang Wang, Junru Chen, Yuhong Ou, Qiuning Zhang, Xiaohu Wang

**Affiliations:** 1 The First School of Clinical Medicine, Lanzhou University, Lanzhou, China; 2 Institute of Modern Physics, Chinese Academy of Sciences, Lanzhou, China; 3 Clinical Medical College & Affiliated Hospital of Chengdu University, Chengdu, China; 4 Department of Postgraduate, University of Chinese Academy of Sciences, Beijing, China; 5 Heavy Ion Therapy Center, Lanzhou Heavy Ions Hospital, Lanzhou, China; 6 Gansu University of Chinese Medicine, Lanzhou, China

**Keywords:** osteosarcoma, PARP inhibitor, olaparib, X-rays, carbon ion, radiosensitization

## Abstract

**Objective:** Osteosarcomas are derived from bone-forming mesenchymal cells that are insensitive to radiation. This study aimed to investigate the radiosensitization of osteosarcoma cells (U2OS and K7M2) using the PARP inhibitor olaparib combined with X-rays or carbon ions (C-ions).

**Methods:** The effect of olaparib on the proliferation of osteosarcoma cells after irradiation was assessed using CCK-8 and clone formation assays. Cells were treated with olaparib and/or radiation and the effects of olaparib on the cell cycle and apoptosis were analysed by flow cytometry after 48h. Immunofluorescence was used to stain the nuclei, γ-H2AX, 53BP1, and Rad51 proteins, and the number of γ-H2AX, 53BP1, and Rad51 foci was observed under a fluorescence microscope. The effect of olaparib combined with radiation on double-stranded DNA breaks in osteosarcoma cells was evaluated.

**Results:** At the same radiation dose, olaparib reduced the proliferation and colony formation ability of irradiated osteosarcoma cells (P < 0.05). Olaparib monotherapy induced minimal apoptotic effects and G_2_/M phase arrest in osteosarcoma cells and irradiation alone induced moderate apoptosis and G_2_/M phase arrest. However, radiation combined with olaparib significantly increased the percentage of apoptotic cells and G2/M phase arrest in osteosarcoma cells (P < 0.05). Immunofluorescence experiments showed that compared to the radiation group, the formation of γ-H2AX and 53BP1 foci was significantly increased in the combined group (P < 0.05). The expression levels of Rad51 foci in the irradiated group were higher than those in the control group (P < 0.05). However, the number of Rad51 foci in the combined group was significantly decreased (P < 0.05).

**Conclusion:** The PARP inhibitor olaparib combined with irradiation (X-rays or C-ions) enhanced the radiosensitivity of osteosarcoma cell lines (U2OS and K7M2). Our findings provide a potential theoretical basis for the clinical application of olaparib in overcoming radiation resistance in osteosarcoma.

## Introduction

Osteosarcoma is derived from bone-forming mesenchymal cells and is the most common malignant tumour of the bones, especially in children and adolescents 1-3. The comprehensive treatment of osteosarcoma based on surgery, chemotherapy, and radiotherapy has gradually become a consensus in China, Europe, and North America 4. However, surgical resection of some osteosarcomas adjacent to complex structures, such as the pelvis and axis, is difficult to achieve 4-6.

Based on previous results of conventional photon radiotherapy (RT), sarcomas are considered insensitive to radiation 7 because higher doses (> 70 Gy) are difficult to achieve using photon RT techniques 8, 9. Carbon ions RT (C-ions RT) is an advanced RT technique that is increasingly utilised in Asia and Europe 10. The excellent physical advantages (Bragg peak effect) of C-ions RT provide better protection for normal tissues. In addition, C-ions RT has a higher relative biological effectiveness (RBE), which is especially beneficial for radio-resistant tumours, such as osteosarcoma 10-12. In our previous systematic review of C-ions RT for osteosarcoma, the local control (LC) rates at 2, 3, and 5 years were 73.1%, 69.2%, and 61.5%, respectively 10. Although the LC rates of C-ions RT were significantly improved compared to those of photons, the failure modes were still local recurrence and distant metastasis 10. Matsunobu et al. reported that in 78 patients with unresectable trunk osteosarcomas who received C-ions RT, the rates of local recurrence and distant metastases were 26.9% and 52.6%, respectively 13. Therefore, the identification of suitable radiosensitisers may be an effective way to improve local control and reduce recurrence or metastasis.

Radiation can cause DNA damage in cells through direct and indirect action, resulting in single-strand breaks (SSBs) and double-strand breaks (DSBs). Unrepaired DSBs can lead to cell death 14, 15. Poly (ADP-ribose) polymerases (PARP) are DNA repair enzymes that are widely expressed in the nuclei of mammalian cells and are involved in several DNA repair pathways, including SSB repair, DSB repair, and chromatin remodeling 16-19. Inhibition of PARP leads to the increased accumulation of DNA damage and enhanced cytotoxicity. PARP inhibitors (PARPi) have the potential to increase radiation damage, which has been reported in melanoma, gynecologic cancer, chondrosarcoma, glioblastoma high-grade serous ovarian carcinomas, and hypopharyngeal cancer 20-25. Moreover, tumour cells with deficient homologous recombination (HR) repair mechanisms, including BRCA1/2-mutated tumours and BRCA-like tumours (tumours with HR-related gene mutations except for BRCA1/2 mutations—i.e., BRCAness), are more sensitive to PARPi due to the "synthetic lethality effect" 26, 27.

Based on exome sequencing, osteosarcoma may exhibit a specific genome instability signature characteristic of a BRCA deficiency or BRCA-like tumours 28. Olaparib (originally called AZD2281) is a pan-PARP inhibitor commonly used to treat BRCA1/2-mutated or BRCA-like tumours 26, 27. This study aimed to reveal the radiosensitizing effect of olaparib when combined with X-rays or C-ions irradiation in osteosarcoma cell lines, which may improve the therapeutic efficacy of radiotherapy on osteosarcoma.

## Materials and Methods

### Cell cultures

The human osteosarcoma cell line U2OS (HTB-96) and murine osteosarcoma cell line K7M2 (CRL-2836) were purchased from ATCC. The two osteosarcoma cell lines were cultured in DMEM (HyClone^TM^, Logan, UT) with 10% (v/v) foetal bovine serum (FBS; HyClone^TM^, Logan, UT, USA), 100 U/ml penicillin, and 100 mg/ml streptomycin (Life Technologies). All cells were cultured in an incubator with a constant temperature of 37 ^◦^C and a humidified 5% CO_2_ atmosphere.

### Irradiation condition

For X-rays irradiation, a photon beam was delivered using an X-RAD225 OptiMAX irradiator (Precision X-Ray, USA). The ray parameters were as follows: a tube tension of 225 kV and a corresponding dose rate of 2 Gy/min).

The C-ions beam was obtained from a heavy-ion medical machine (HIMM) at Lanzhou Heavy Ion Hospital. The ray parameters are as follows: energy of 120 MeV/u, with an ion linear energy transfer (LET) of 80 keV/μm at the centre of the spread-out Bragg peak, and a dose rate of 2 Gy/min.

### Exome gene sequencing of cell lines

Exome sequencing was performed on the U2OS and K7M2 cell lines. Mutations in DNA repair gene sequences, especially homologous recombinations, were analysed. After obtaining the Sequenced Reads, information analysis procedures, including sequencing data quality assessment, mutation detection, mutation annotation, and statistics were performed in the presence of reference sequences or reference genomes (GRCm37/mm9). Bioinformatics analysis were performed using the FASTP v0.12.4 software for data filtering and quality control. BWA-MEM2 v2.2.0 was then used for alignment; the GATK v4.2.2.0 pipeline was used to produce BAM files, and Mosdepth v0.2.9 was used for quality control of the BAM files, according to the Broad Institute recommendations.

### Bioinformatic analysis

Data on osteosarcoma were obtained from TARGET datebase. According to the median expression levels of PARP1/2, the osteosarcoma samples were divided into high-expression and low-expression groups. The Gene Set Enrichment Analysis (GSEA) was conducted for these data. The enriched results were considered as significant that satisfied the following criteria simultaneously: 1) |NES| ˃ 1.0, 2) Nominal *p* < 0.05, 3) FDR *q* < 0.05.

### PARPi treatment

Olaparib powder (M1664; AbMole, USA) was dissolved in DMSO (10 mM) and diluted to the desired concentration with DMEM. Negative control samples were treated with the same DMSO concentration as that of the test sample.

### Cell counting kit-8 (CCK-8) assay

Cell proliferation was determined using the cell count kit-8 assay (CCK-8; Sunview Technology, Shenzhen, China). The U2OS and K7M2 cells were counted using an automatic cell counter (LUNA-II; Logo Biosystems, Korea). Next, U2OS (200μL: approximately 4 × 10^3^ cells) and K7M2 (200μL: approximately 4 × 10^3^ cells) cells were added to each well (six replicates per group) of 96-well plates. The 96-well plates were incubated overnight (37°C, 5% CO_2_) to allow cell adherence. Cell proliferation was detected at different times (0, 12, 24, 48, 72h), different olaparib concentrations (0, 2.5, 5, 10, 20, 40, 80 μM) and different irradiation doses (0, 1, 2, 4 Gy). Each well was inoculated with 20 μL CCK-8 and incubated for 2 h (37°C, 5% CO_2_). The optical density (OD) values were measured at 450 nm using a microplate reader (Tecan Infinite 200M, Switzerland).

### Colony formation assay

U2OS and K7M2 cells were counted using an automatic cell counter (LUNA-II; Logo Biosystems, Korea). An appropriate number of cells were seeded into six-well plates (U2OS_X-rays_: 0 Gy group: 300 cells/well, 1 Gy group: 300 cells/well, 2 Gy group: 400 cells/well, 4 Gy group: 800 cells/well; U2OS_C-ions_: 0 Gy group: 300 cells/well, 1 Gy group: 600 cells/well, 2 Gy group: 1200 cells/well, 4 Gy group: 6000 cells/well) and 60-mm dishes (K7M2_X-rays_: 0 Gy group: 200 cells/well, 1 Gy group: 200 cells/well, 2 Gy group: 400 cells/well, 4 Gy group: 1600 cells/well; K7M2_C-ions_: 0 Gy group: 200 cells/well, 1 Gy group: 400 cells/well, 2 Gy group: 1200 cells/well, 4 Gy group: 6000 cells/well). After overnight incubation at 37°C, 5% CO_2_, the cells were irradiated with X-rays and C-ions, respectively. After 10-14 days of culture, the cells were fixed and stained for 15 min. Colonies were observed and counted under a microscope. Plating efficiency (PE) was calculated as colony number/inoculation number × 100% while survival fraction (SF) was calculated as the colony rate in the experimental group/colony rate in the control group × 100%. The cell dose-survival curve was generated using the GraphPad Prism 6 software using the following formula: SF=e^(-(αD + βD^2^)). This experiment were carried out independent 3 times.

### Cell migration

The cell migration assay was used to describe the migration behavior of steosarcoma cells. Cells were seeded and incubated for 24 hours at 37 °C, generating monolayers in the cavities of cell culture plates. To provoke starvation, the incubation medium was replaced by serum-reduced cell culture medium (2% FBS). After another 24 hours, the monolayer in each cavity was scratched with a 200 μl pipet tip, creating precise cell-free wounds. To record migration, images of the same scratch area were repeatedly taken by a microscope after 48 hours of irradiation. The varying area of the cell wound over time was measured by an image analyzing ImageJ software. This experiment were carried out independent 3 times.

### Cell apoptosis assay

An annexin V-FITC/PI apoptosis detection kit (Yeasen Biotechnology, Shanghai, China) was used to detect the occurrence of early and late apoptosis. Trypsin without ethylenediaminetetraacetic acid (EDTA) was used to detach the cells. The single-cell suspension was incubated with 150 μL binding buffer, 5 μL FITC Annexin V, and 10 μL propyl iodide (PI) at room temperature for 15 minutes. At least 10,000 cells were collected from each sample and were analysed using the FlowSight flow cytometer (Amnis, Seattle, WA). The IDEAS software (v6.0) was used to analyse apoptosis data. This experiment were carried out independent 3 times.

### Cell cycle assay

U2OS and K7M2 cells were detached using trypsin and fixed overnight with 75% ethanol at -20 °C. The fixed cells were centrifuged to remove the ethanol and were incubated in 3 mL PBS for 15 min to rehydrate the cells. The cells were re-centrifuged and the supernatant was removed. Single cells were resuspended in 150 μL DNA Staining solution (MultiSciences, Hangzhou, China) and incubated in a dark place for 30 min at room temperature. At least 20,000 cells were collected from each sample and their fluorescence was immediately analysed using the FlowSight flow cytometer (Amnis, Seattle, WA, USA). This experiment were carried out independent 3 times.

### Immunofluorescence staining of γ-H2AX, 53BP1, and Rad51

Cells were attached to 20-mm immunofluorescence culture dishes (NEST Biotechnology, Jiangsu, China). The cells were fixed with 4% fixative solution 2 h and 12 h after irradiation, permeabilised with 0.5% Triton X-100, and subsequently blocked using 10% bovine serum albumin (BSA) in TBST. Cells were incubated overnight at 4^◦^C with primary antibodies against γ-H2AX (1:200) (ab26350; Abcam), 53BP1 (1:200) (ab175933; Abcam), and Rad51 (1:200) (ab133534; Abcam). The cells were then incubated with a fluorescent secondary antibody (Immunoway, Beijing, China) in a dark room for 1 h, and then washed three times with TBST. Finally, DAPI (Vector Laboratories, Burlingame, CA, USA) was added for 5 min to label the nuclei. At least 100 cells were viewed, captured, and quantitatively analysed in each immunofluorescence dish using a Zeiss LSM‐700 confocal microscope.

### Western blot analysis

Different treatment groups were irradiated and cultured for 12 hours. Proteins were extracted at specified time points from the cells using RIPA buffer (Solarbio, Beijing, China) supplemented with phenylmethanesulfonyl fluoride (PMSF) and phosphatase inhibitors and quantified using the BCA kit (Solarbio, Beijing, China). The proteins were separated by sodium dodecyl sulfate-polyacrylamide gel electrophoresis (SDS-PAGE) and transferred to polyvinylidene fluoride membranes (Immobilon-P, Ireland). After blocking in 5% skimmed milk for 2 hours at room temperature, the membranes were incubated overnight at 4℃ with the primary antibodies γ‐H2AX (1:1000), 53BP1 (1:1000), Rad51 (1:1000), and β-Actin (1:2000) (Cat No. 66009-1-lg; Proteintech). and then incubated with secondary antibodies at room temperature for 1 hour. Bands were visualized with a chemiluminescence imaging system (QuickChemi5200, Monad, China). We used the ImageJ software to quantify the relative protein expression. Meanwhile, the β-Actin levels were used to normalize the results. This experiment were carried out independent 3 times.

### Statistical analysis

All experiments were repeated at least thrice. All results were analysed using a two-sided Mann-Whitney U test using GraphPad Prism 9.0 (La Jolla, CA, USA). Data are presented as the means ± SD. Statistical significance was set at P < 0.05 (*). Figures were plotted using GraphPad Prism 9.0 (La Jolla, CA, USA).

## Results

### Genetic characterisation of U2OS and K7M2 osteosarcoma cells

Because tumour cells with deficient homologous repair mechanisms are more sensitive to PARPi, including tumours with BRCA1/2 mutations and BRCAness, we analysed mutations in a panel of 69 genes involved in DNA repair (Supplementary Data 1 and 2). BRCA1/2 gene mutations in U2OS and K7M2 osteosarcoma cell lines are shown in Table 1. According to mutation analysis, genes with mutation frequencies higher than 10% may alter genetic characteristics (by base substitution, frameshift deletion, and frameshift insertion), including (Supplementary Tables 1 and 2): AURKA, BARD1, BRCA1, BRCA2, BRIP1, CHEK1, ERCC2, FANCD2, FANCI, FANCM, NBN, PIK3R2, RAD51D, RNF168, SMARCA2, TDG, TOPBP1, UIMC1, and XRCC3 (U2OS cells); ARID1A, ATM, ATR, ATRX, BABAM1, BLM, BRCA2, BRIP1, ERCC1, FANCA, FANCG, FANCI, FANCL, FANCM, NBN, PIK3R2, RAD51C, RPA1, UIMC1, and XRCC4 (K7M2 cells).

### GSEA analysis

**Figure 1:**
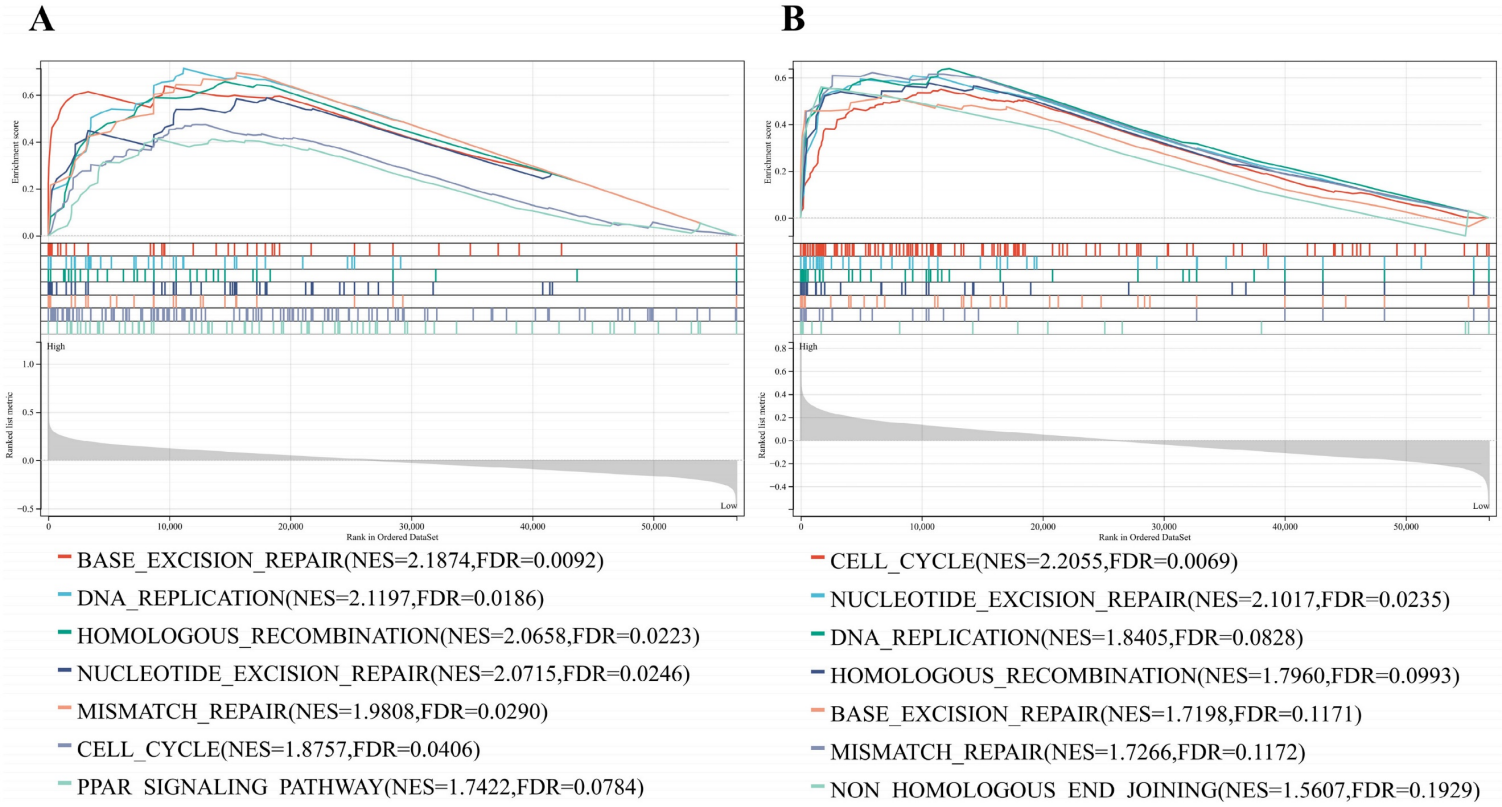
GSEA enrichment analysis of PARP and DNA-related signaling pathway. (A) GSEA enrichment analysis of PARP1 and DNA-related signaling pathway. (B) GSEA enrichment analysis of PARP2 and DNA-related signaling pathway.

Based on the large number of DNA damage repair gene mutations in osteosarcoma cells, we used GSEA analysis was applied to explore the PARP-related signaling pathways. The genes in the PARP1/2 high-expression group were mainly enriched in DNA-related signaling pathways including cell cycle, DNA replication, base excision repair, HR, nucleotide excision repair, mismatch repair, and PARP signaling pathways (Fig. 1 A and B).

### Co-treatment with olaparib and irradiation inhibited the proliferation of osteosarcoma cells

**Figure 2:**
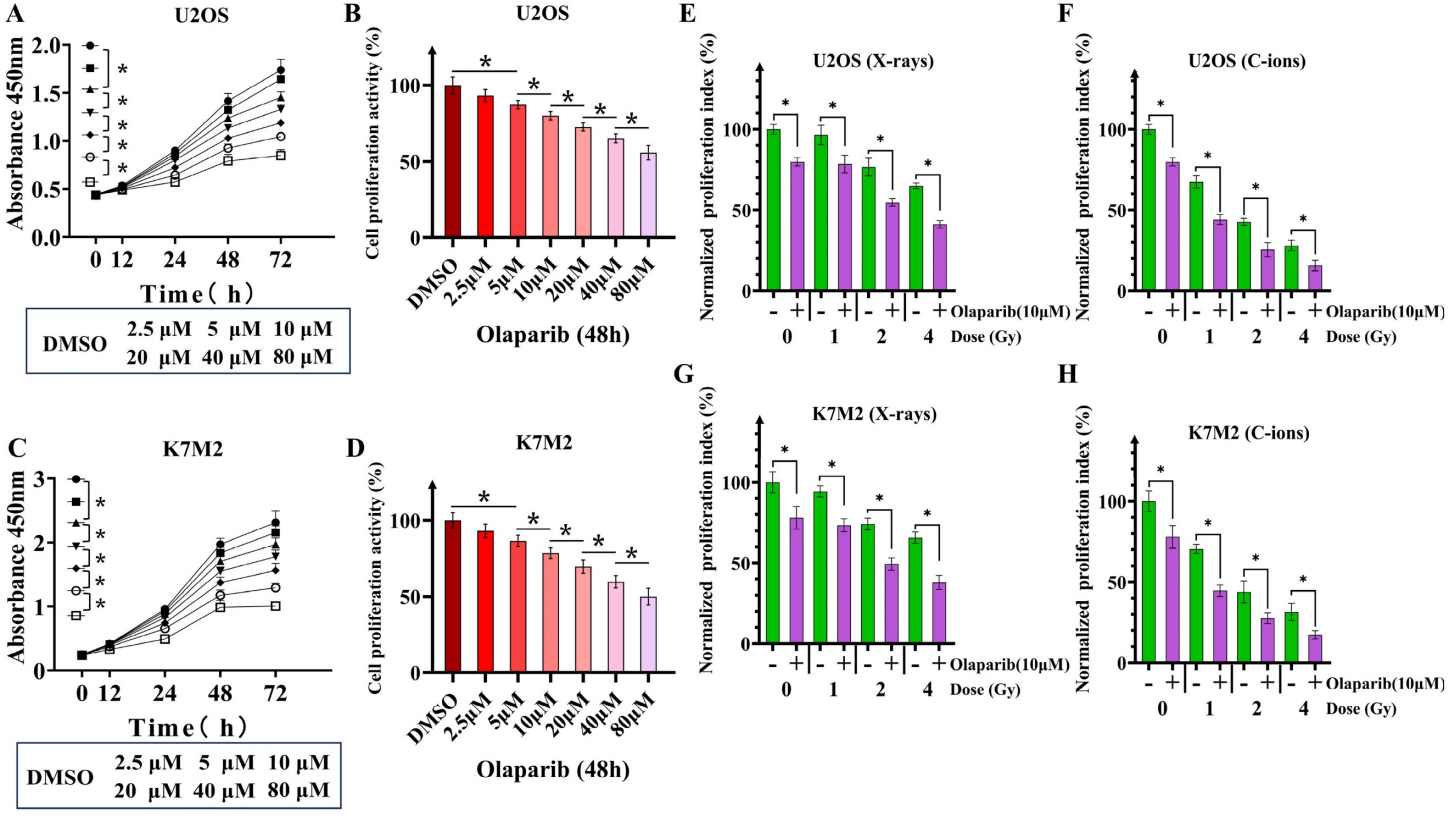
The effect of olaparib on the proliferation of osteosarcoma cells (A-D). Time dependence of olaparib at different concentrations on U2OS (A) and K7M2 (C) osteosarcoma cells. Inhibition of proliferation of U2OS (B) and K7M2 (D) osteosarcoma cells by different concentrations of olaparib after 48 hours. The effect of olaparib combined with irradiation (X-rays or C-ions) on the proliferation of osteosarcoma cells (E-H), DMEM medium containing olaparib (10 μM) was added 2 hours before irradiation and removed 48 hours after irradiation. Co-treatment of X-rays and olaparib significantly inhibited proliferation of U2OS (E) and K7M2 (G) osteosarcoma cells. Co-treatment of C-ions and olaparib significantly inhibited proliferation of U2OS (F) and K7M2 (H) osteosarcoma cells. Cell counting kit-8 (CCK-8) assay were six replicates in each group. Data were considered to be significantly different when *p* < 0.05 (*).

Because U2OS and K7M2 cells have mutations in DNA repair gene sequences, specifically homologous recombination, we observed that olaparib monotherapy inhibited the proliferative activity of osteosarcoma cells (Fig. 2A-D). We evaluated the effect of olaparib on the proliferative activity of irradiated osteosarcoma cells based on the response of non-irradiated cells (Fig. 2E-H). Different concentrations of olaparib had different inhibitory effects on U2OS and K7M2, so we finally selected olaparib concentration with a inhibition rate of 20%. When olaparib concentration was 10μM the treatment time was 48 hours, the cell proliferation activity of U2OS cells was 80.06% ± 2.7%, and that of K7M2 cells was 78.55% ± 3.4% (Fig. 2B and D). Compared to the irradiation group, olaparib further decreased the proliferative activity of irradiated osteosarcoma cells as demonstrated by the CCK-8 assay (*p* < 0.05). Normalized proliferative index of irradiated U2OS osteosarcoma cells by X-rays: U2OS_0Gy_ vs. U2OS_Olaparib_: 100% ± 3.04% vs. 79.80% ± 2.53%; U2OS_1Gy_ vs. U2OS_1Gy+Olaparib_: 96.54% ± 6.02% vs. 78.47% ± 5.45%; U2OS_2Gy_ vs. U2OS_2Gy+Olaparib_: 76.58% ± 5.57% vs. 54.71% ± 2.28%; U2OS_4Gy_ vs. U2OS_4Gy+Olaparib_: 65.03% ± 1.62% vs. 41.08% ± 2.32%. Normalized proliferative index of irradiated U2OS osteosarcoma cells by C-ions: U2OS_0Gy_ vs. U2OS_Olaparib_: 100% ± 3.04% vs. 79.80% ± 2.53%; U2OS_1Gy_ vs. U2OS_1Gy+Olaparib_: 67.39% ± 3.77% vs. 44.08% ± 3.06%; U2OS_2Gy_ vs. U2OS_2Gy+Olaparib_: 42.71% ± 2.14% vs. 25.48% ± 4.38%; U2OS_4Gy_ vs. U2OS_4Gy+Olaparib_: 28.01% ± 3.28% vs. 15.58% ± 3.32%. Normalized proliferative index of irradiated K7M2 osteosarcoma cells by X-rays: K7M2_0Gy_ vs. K7M2_Olaparib_: 100% ± 6.50% vs. 78.00% ± 7.05%; K7M2_1Gy_ vs. K7M2_1Gy+Olaparib_: 94.33% ± 3.48% vs. 73.35% ± 3.92%; K7M2_2Gy_ vs. K7M2_2Gy+Olaparib_: 74.16% ± 3.65% vs. 49.33% ± 3.87%; K7M2_4Gy_ vs. K7M2_4Gy+Olaparib_: 65.74% ± 3.52% vs. 37.91% ± 4.45%. Normalized proliferative index of irradiated K7M2 osteosarcoma cells by C-ions: K7M2_0Gy_ vs. K7M2_Olaparib_: 100% ± 6.50% vs. 78.00% ± 7.05%; K7M2_1Gy_ vs. K7M2_1Gy+Olaparib_: 70.44% ± 2.79% vs. 44.63% ± 3.55%; K7M2_2Gy_ vs. K7M2_2Gy+Olaparib_: 43.84% ± 6.81% vs. 27.54% ± 3.37%; K7M2_4Gy_ vs. K7M2_4Gy+Olaparib_: 31.44% ± 5.26% vs. 17.27% ± 2.64%.

### Olaparib reduces colony formation in irradiated osteosarcoma cells

**Figure 3:**
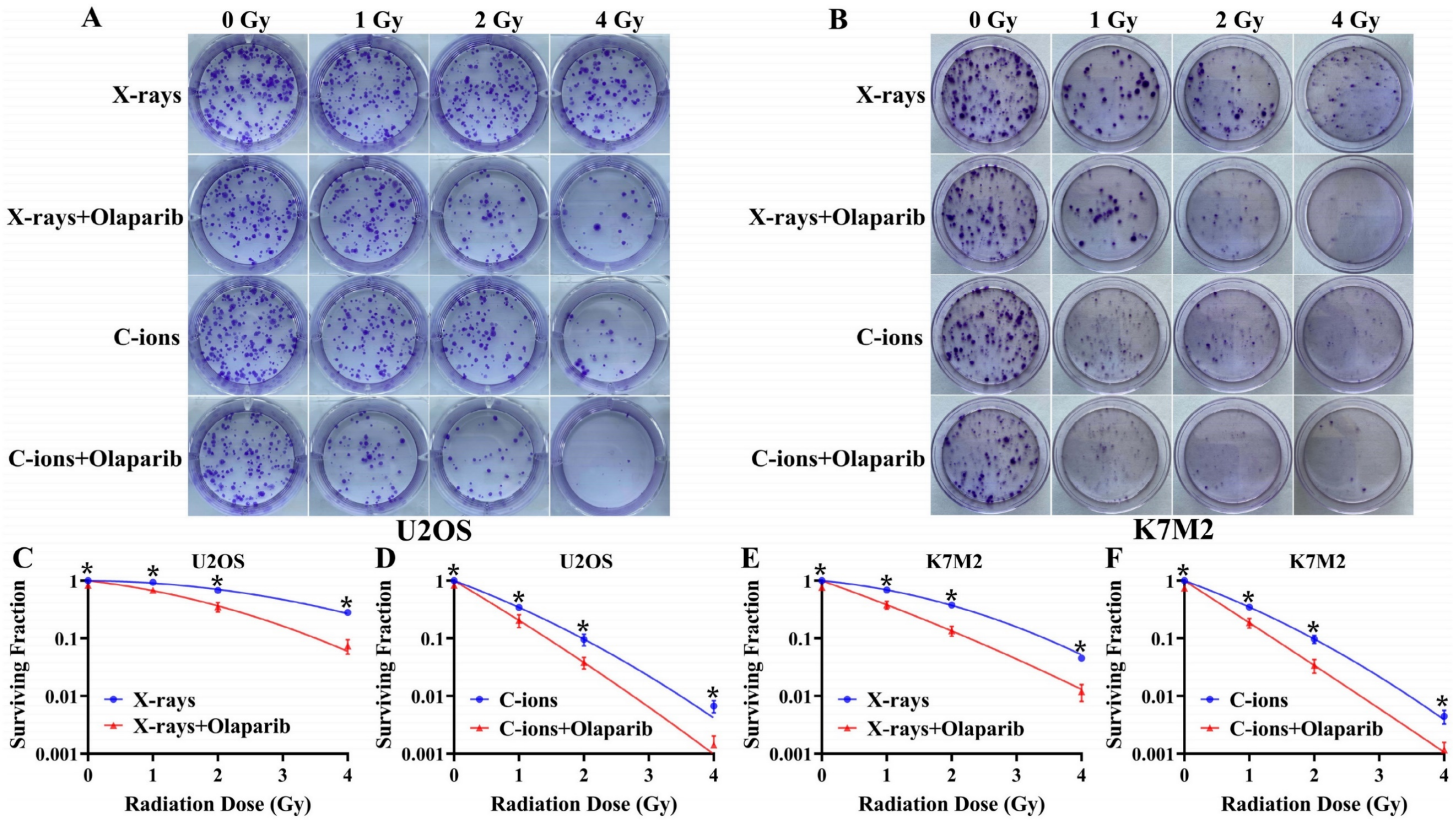
The effect of olaparib combined with irradiation (X-rays or C-ions) on the colony formation of U2OS (A) and K7M2 (B) osteosarcoma cells. DMEM medium containing olaparib (10 μM) was added 2 hours before irradiation and removed 48 hours after irradiation. (C) U2OS cells were irradiated with X-rays (blue line) or X-rays with olaparib (red line). (D) U2OS cells were irradiated with C-ions (blue line) or C-ions with olaparib (red line). (E) K7M2 cells were irradiated with X-rays (blue line) or X-rays with olaparib (red line). (F) K7M2 cells were irradiated with C-ions (blue line) or C-ions with olaparib (red line). Cell survival (%) were shown as mean +/- SD of 3 independent experiments performed in triplicate for X-rays and C-ions. For each dose, clonogenic survival were considered to be significantly different (with and without olaparib) when *p* < 0.05 (*).

As shown in Fig. 3, olaparib reduced colony formation in irradiated osteosarcoma cells (Fig. 3A and B). At the same radiation dose (For example, in 2Gy), clonogenic survival in the combination group was markedly lower than the irradiation group (*p* < 0.05): U2OS_X-rays_: 35.26% ± 7.99% vs. 67.8% ± 3.87%; U2OS_C-ions_: 3.81 ± 1.03% vs. 9.60% ± 2.68%; K7M2_X-rays_: 13.54% ± 3.25% vs. 37.90% ± 3.46%; K7M2_C-ions_: 3.40% ± 1.12% vs. 9.71% ± 1.88% (Fig. 3C-F). The enhancement ratio (ER) was calculated for X-rays and C-ions using the D10 and D37 values (Table 2). The radiosensitization ability of olaparib to C-ions was stronger than X-rays for osteosarcoma: the ER of U2OS for X-rays and C-ions were 1.42 and 1.55, respectively; the ER of K7M2 for X-rays and C-ions were 1.63 and 1.73, respectively (Table 2).

### Cell migration

**Figure 4:**
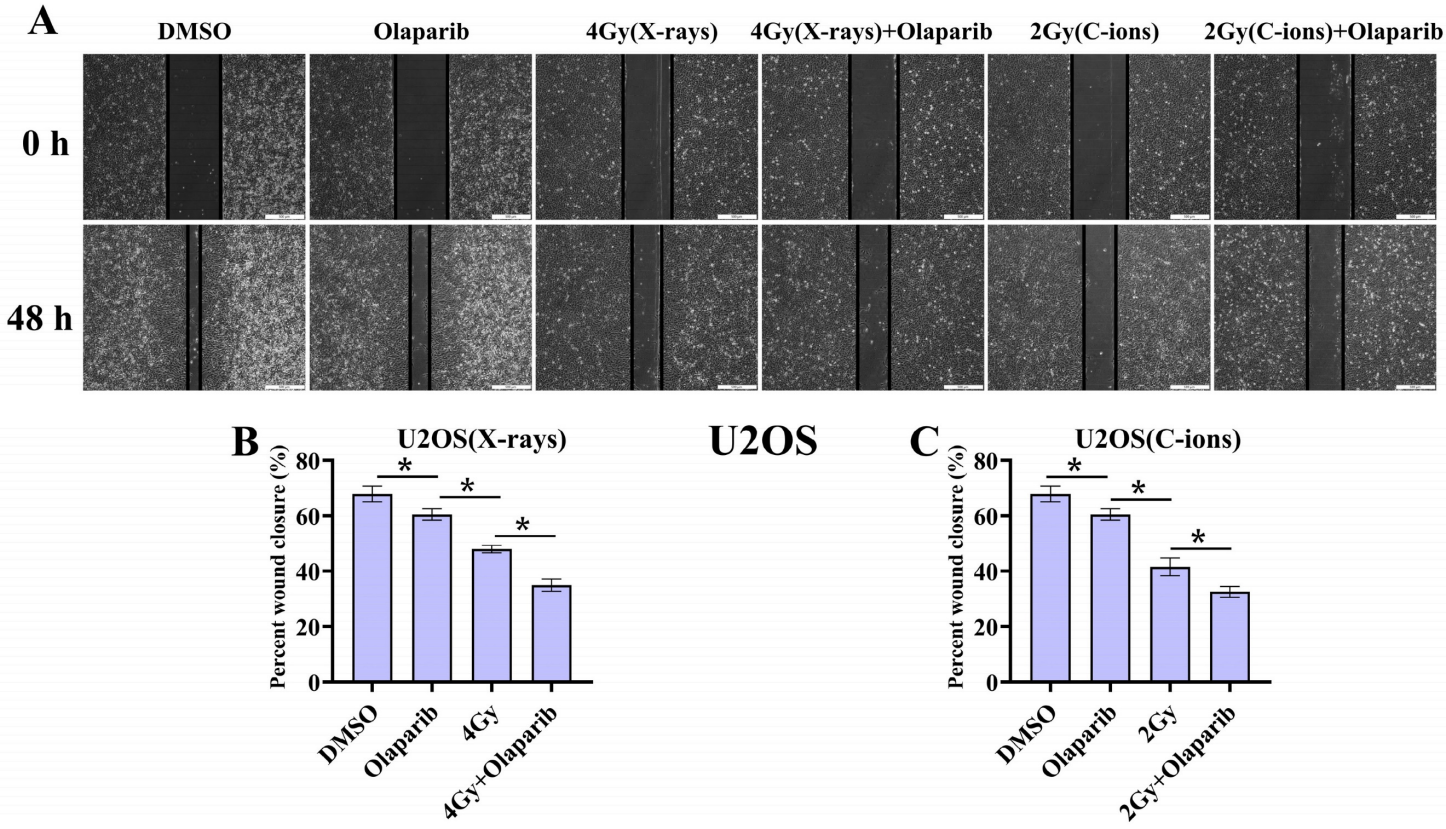
The effect of olaparib combined with irradiation (X-rays or C-ions) on the cell migration of U2OS osteosarcoma cells (A). DMEM medium containing olaparib (10 μM) was added 2 hours before irradiation and analyzed area of the cell wound by 48 hours after irradiation. (B) Percent wound closure (%) of olaparib combined with X-rays. (C) Percent wound closure (%) of olaparib combined with C-ions. Percent wound closure (%) were shown as mean +/- SD of 3 independent experiments performed in triplicate for X-rays and C-ions. Data were considered to be significantly different when *p* < 0.05 (*).

**Figure 5:**
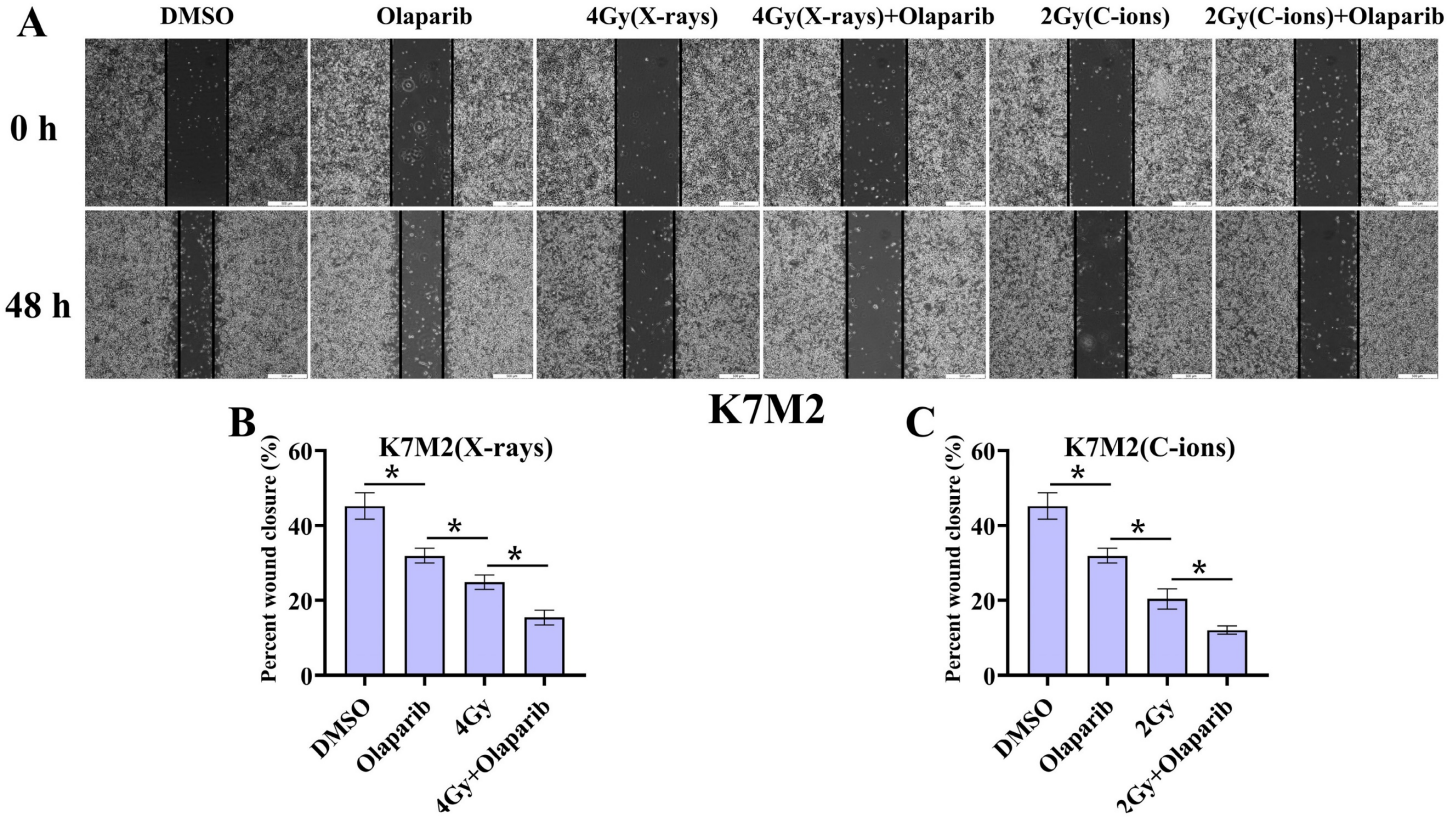
The effect of olaparib combined with irradiation (X-rays or C-ions) on the cell migration of K7M2 osteosarcoma cells (A). DMEM medium containing olaparib (10 μM) was added 2 hours before irradiation and analyzed area of the cell wound by 48 hours after irradiation. (B) Percent wound closure (%) of olaparib combined with X-rays. (C) Percent wound closure (%) of olaparib combined with C-ions. Percent wound closure (%) were shown as mean +/- SD of 3 independent experiments performed in triplicate for X-rays and C-ions. Data were considered to be significantly different when *p* < 0.05.

As shown in Fig. 4 and Fig. 5, our results showed that the cell migration of all treatment groups were lower than those in the control group (*p* < 0.05) (Fig. 4 and 5). In addition, the data showed that olaparib combined irradiation group significantly inhibited cell migration compared to that in the irradiated-only U2OS and K7M2 osteosarcoma cells (*p* < 0.05): U2OS_X-rays_: 34.96% ± 2.27% vs. 48.00% ± 1.33%; U2OS_C-ions_: 32.57 ± 1.95% vs. 41.58% ± 3.21%; K7M2_X-rays_: 15.45% ± 1.97% vs. 24.88% ± 1.93%; K7M2_C-ions_: 12.09 ± 1.12% vs. 20.44% ± 2.70% (Fig. 4 and 5).

### Olaparib induces apoptosis in irradiated osteosarcoma cells

**Figure 6:**
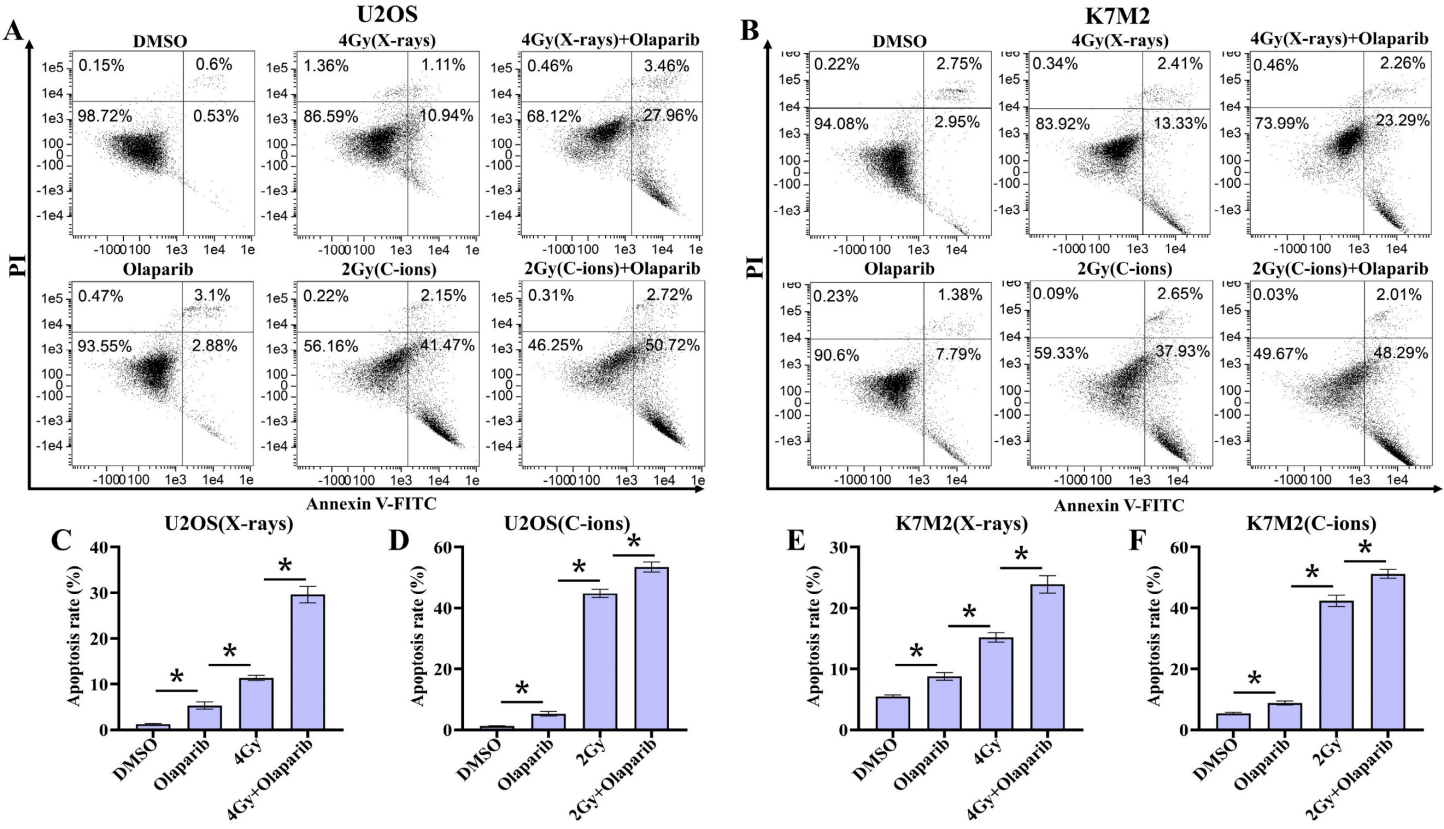
The effect of olaparib combined with irradiation (X-rays or C-ions) on the apoptosis rate of U2OS (A) and K7M2 (B) osteosarcoma cells. DMEM medium containing olaparib (10 μM) was added 2 hours before irradiation and analyzed by flow cytometry 48 hours after irradiation. (C) U2OS cells were irradiated with X-rays or X-rays with olaparib. (D) U2OS cells were irradiated with C-ions or C-ions with olaparib. (E) K7M2 cells were irradiated with X-rays or X-rays with olaparib. (F) K7M2 cells were irradiated with C-ions or C-ions with olaparib. Apoptosis rate (%) were shown as mean +/- SD of 3 independent experiments performed in triplicate for X-rays and C-ions. Data were considered to be significantly different when *p* < 0.05 (*).

Since targeting cellular DNA damage repair pathways may induce apoptosis or death of tumor cells, we evaluated the effect of olaparib induces apoptosis in irradiated osteosarcoma cells. Apoptosis in osteosarcoma cells was analysed by flow cytometry after Annexin V-FITC/PI staining (Fig. 6A and B). Our results showed that the apoptosis rates of all treatment groups were higher than those in the control group (*p* < 0.05) (Fig. 6C-F). In addition, the data showed that olaparib significantly enhanced X-rays and C-ions induced apoptosis compared to that in the irradiated-only U2OS and K7M2 osteosarcoma cells (*p* < 0.05): U2OS_X-rays_: 29.62% ± 1.80% vs. 11.39% ± 0.57%; U2OS_C-ions_: 53.46 ± 1.64% vs. 44.80% ± 1.37%; K7M2_X-rays_: 23.92% ± 1.42% vs. 15.88% ± 0.76%; K7M2_C-ions_: 51.23 ± 1.51% vs. 42.35% ± 1.85% (Fig. 6C-F).

### Olaparib induces G_2_/M arrest in irradiated osteosarcoma cells

**Figure 7:**
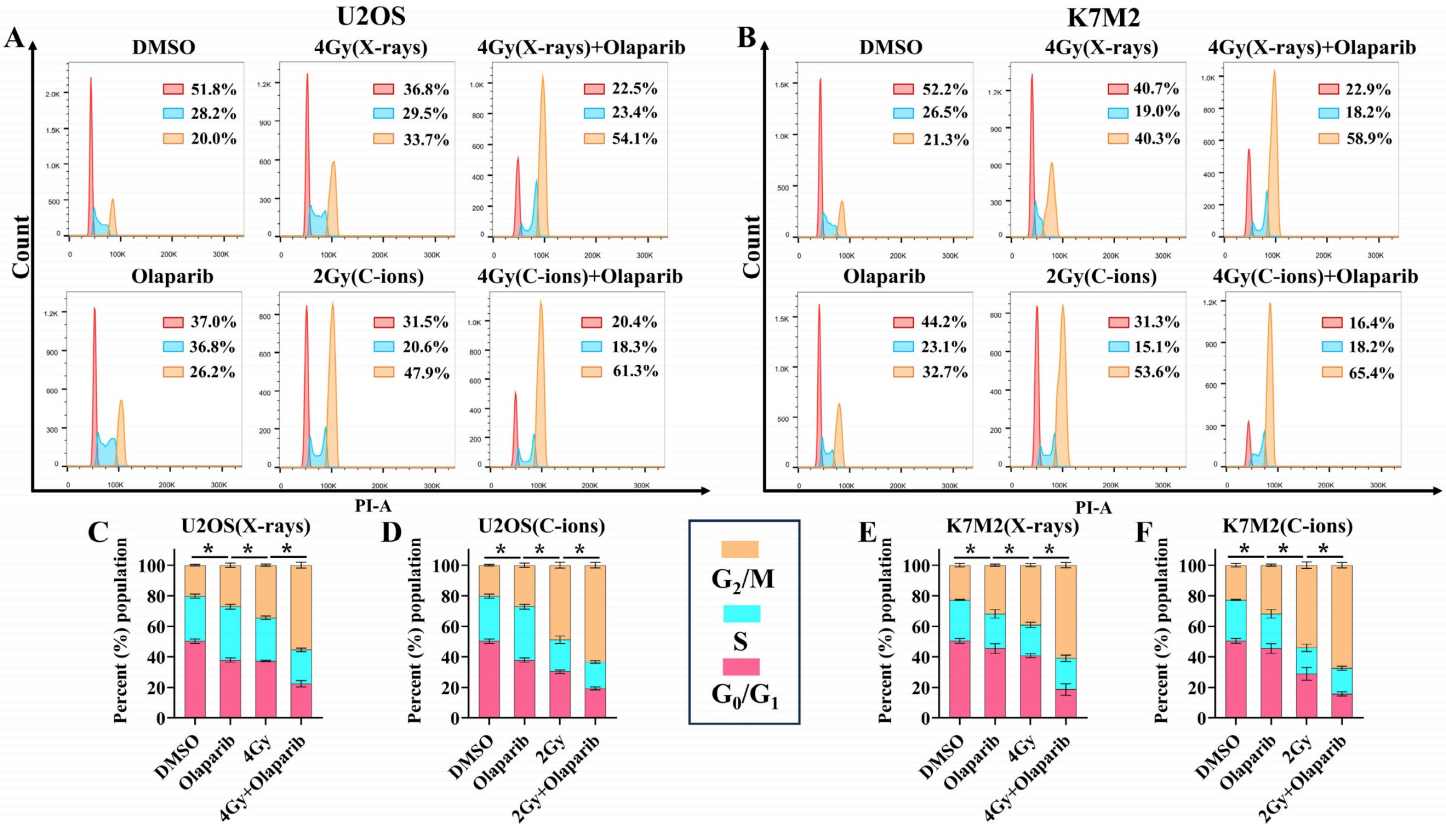
The effect of olaparib combined with irradiation (X-rays or C-ions) on the cell cycle of U2OS (A) and K7M2 (B) osteosarcoma cells. DMEM medium containing olaparib (10 μM) was added 2 hours before irradiation and analyzed by flow cytometry 48 hours after irradiation. (C) U2OS cells were irradiated with X-rays or X-rays with olaparib. (D) U2OS cells were irradiated with C-ions or C-ions with olaparib. (E) K7M2 cells were irradiated with X-rays or X-rays with olaparib. (F) K7M2 cells were irradiated with C-ions or C-ions with olaparib. Percent (%) population were shown as mean +/- SD of 3 independent experiments performed in triplicate for X-rays and C-ions. For treated group, percent (%) population of G_2_/M phase arrest were considered to be significantly different when *p* < 0.05 (*).

GSEA analysis showed that PARP1/2 was associated with osteosarcoma cell cycle, and we evaluated the effect of olaparib on the cell cycle of osteosarcoma cells. The cell cycle distribution was further analysed by flow cytometry after DNA Staining (Fig. 7A and B). As shown in Fig. 7, osteosarcoma cells displayed significant G_2_/M phase arrest in the olaparib, irradiation, and combination groups compared to that in the control group (*p* < 0.05): U2OS_X-rays_: 55.43% ± 1.97% vs. 34.43% ± 0.75%; U2OS_C-ions_: 63.40 ± 1.87% vs. 48.77% ± 1.95%; K7M2_X-rays_: 60.93% ± 1.77% vs. 39.10% ± 1.06%; K7M2_C-ions_: 67.40 ± 1.83% vs. 54.03% ± 2.18% (Fig. 7C-F).

### Olaparib induces DNA damage in irradiated osteosarcoma cells

**Figure 8:**
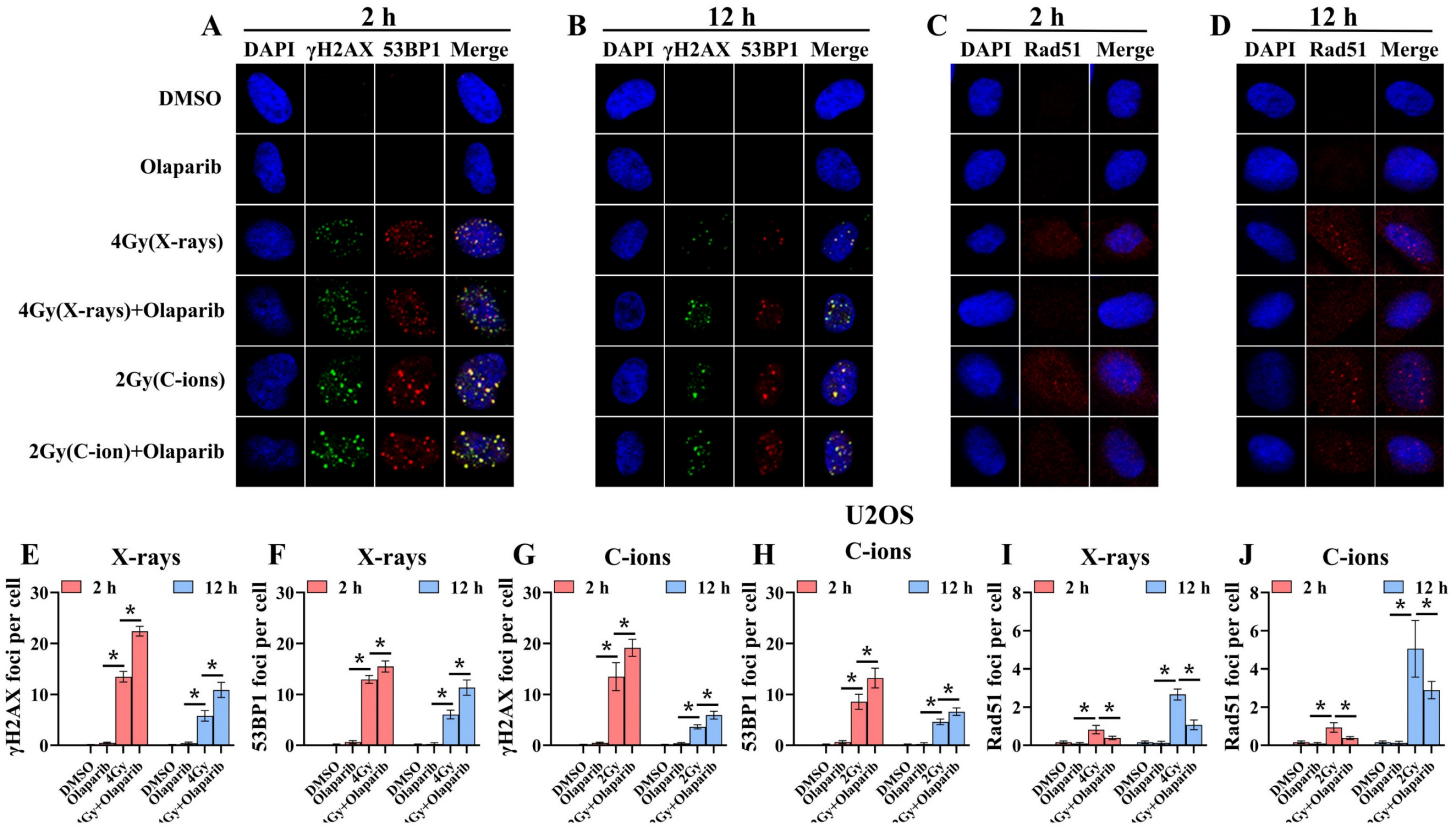
Confocal microscopy views of U2OS osteosarcoma cells with γ‐H2AX/53BP1 (A and B) and Rad51 (C and D) foci formation after X‐rays and C‐ions irradiation over the respective time courses. DMEM medium containing olaparib (10 μM) was added 2 hours before irradiation. (E) Mean number of γ‐H2AX foci per cell after X‐rays irradiation for U2OS cells. (F) Mean number of 53BP1 foci per cell after X‐rays irradiation for U2OS cells. (G) Mean number of γ‐H2AX foci per cell after C‐ions irradiation for U2OS cells. (H) Mean number of 53BP1 foci per cell after C‐ions irradiation for U2OS cells. (I) Mean number of Rad51 foci per cell after X‐rays irradiation for U2OS cells. (J) Mean number of Rad51 foci per cell after C‐ions irradiation for U2OS cells. Mean number of foci per cell were shown as mean +/- SD. Data were considered to be significantly different when *p* < 0.05 (*).

**Figure 9:**
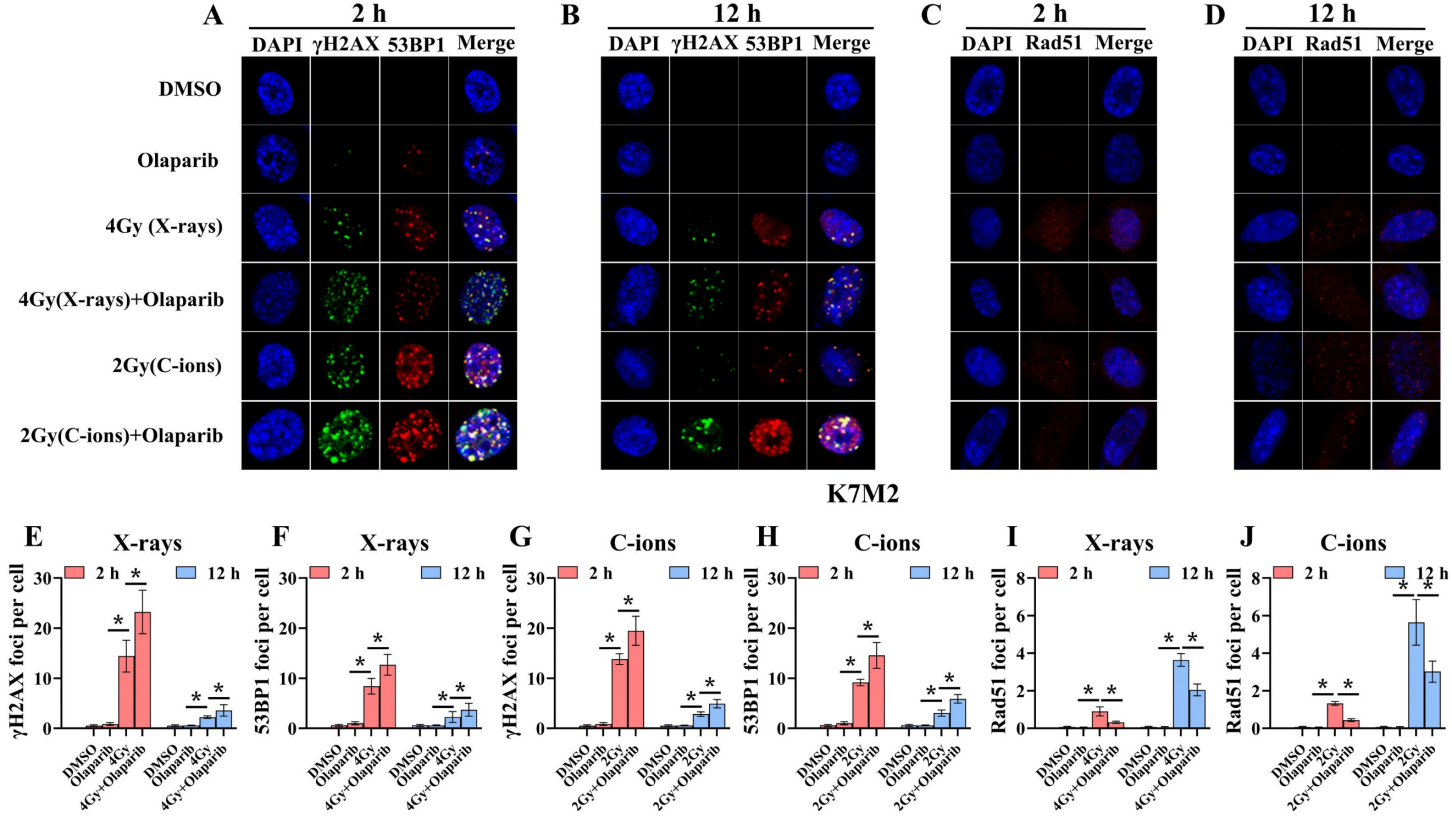
Confocal microscopy views of K7M2 osteosarcoma cells with γ‐H2AX/53BP1 (A and B) and Rad51 (C and D) foci formation after X‐rays and C‐ions irradiation over the respective time courses. DMEM medium containing olaparib (10 μM) was added 2 hours before irradiation. (E) Mean number of γ‐H2AX foci per cell after X‐rays irradiation for K7M2 cells. (F) Mean number of 53BP1 foci per cell after X‐rays irradiation for K7M2 cells. (G) Mean number of γ‐H2AX foci per cell after C‐ions irradiation for K7M2 cells. (H) Mean number of 53BP1 foci per cell after C‐ions irradiation for K7M2 cells. (I) Mean number of Rad51 foci per cell after X‐rays irradiation for K7M2 cells. (J) Mean number of Rad51 foci per cell after C‐ions irradiation for K7M2 cells. Mean number of foci per cell were shown as mean +/- SD. Data were considered to be significantly different when *p* < 0.05 (*).

**Figure 10:**
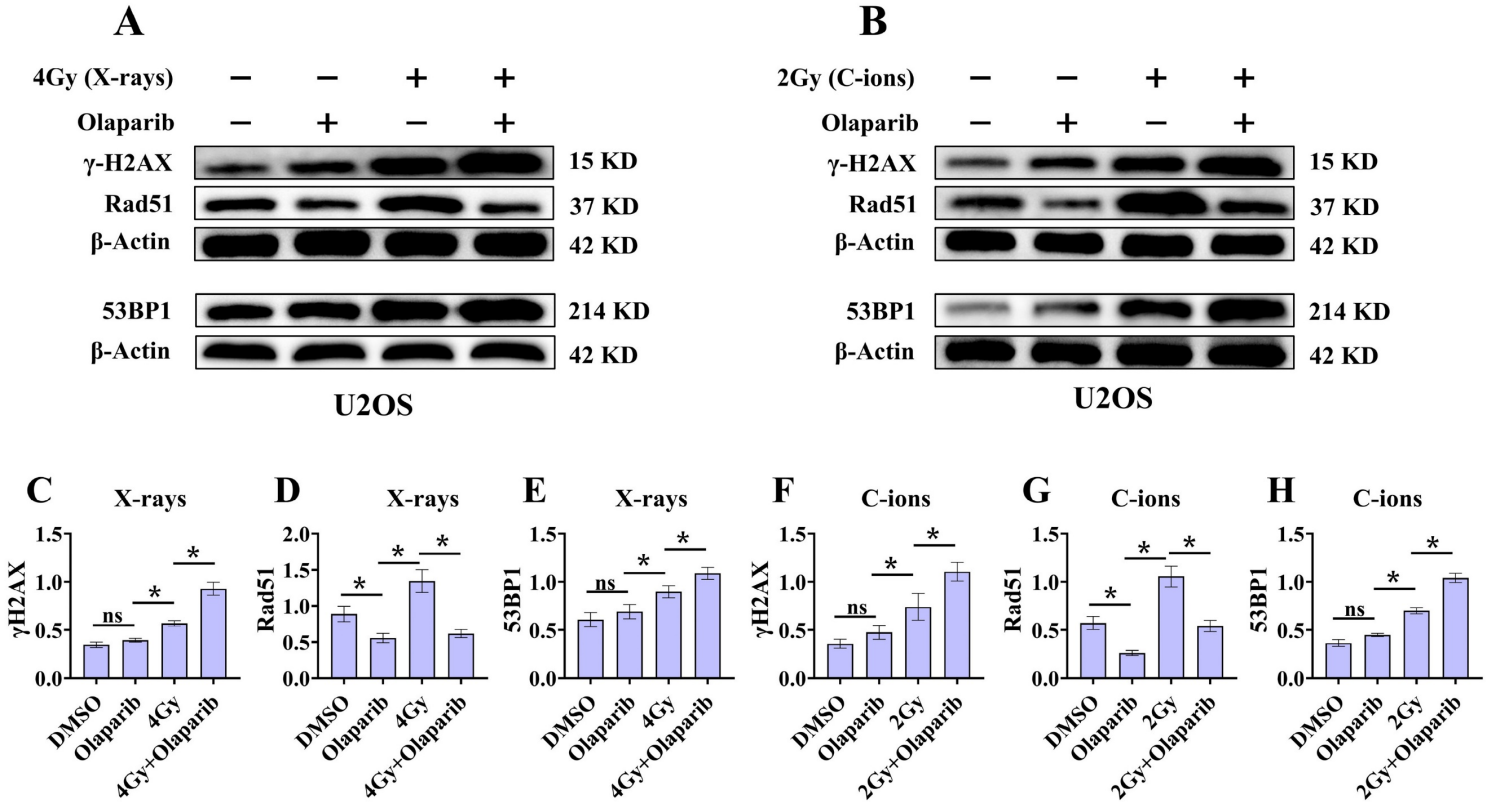
Effect of olaparib on proteins expression of γ‐H2AX, 53BP1, and Rad51 in irradiated U2OS (A and B) osteosarcoma cell. DMEM medium containing olaparib (10 μM) was added 2 hours before irradiation and western blot assay 12 hours after irradiation. (A) Proteins expression of γ‐H2AX, 53BP1, and Rad51 after X‐rays irradiation for U2OS cells. (B) Proteins expression of γ‐H2AX, 53BP1, and Rad51 after C‐ions irradiation for U2OS cells. The relative expression of the proteins (C-H) were shown as mean +/- SD. Data were considered to be significantly different when *p* < 0.05 (*).

**Figure 11:**
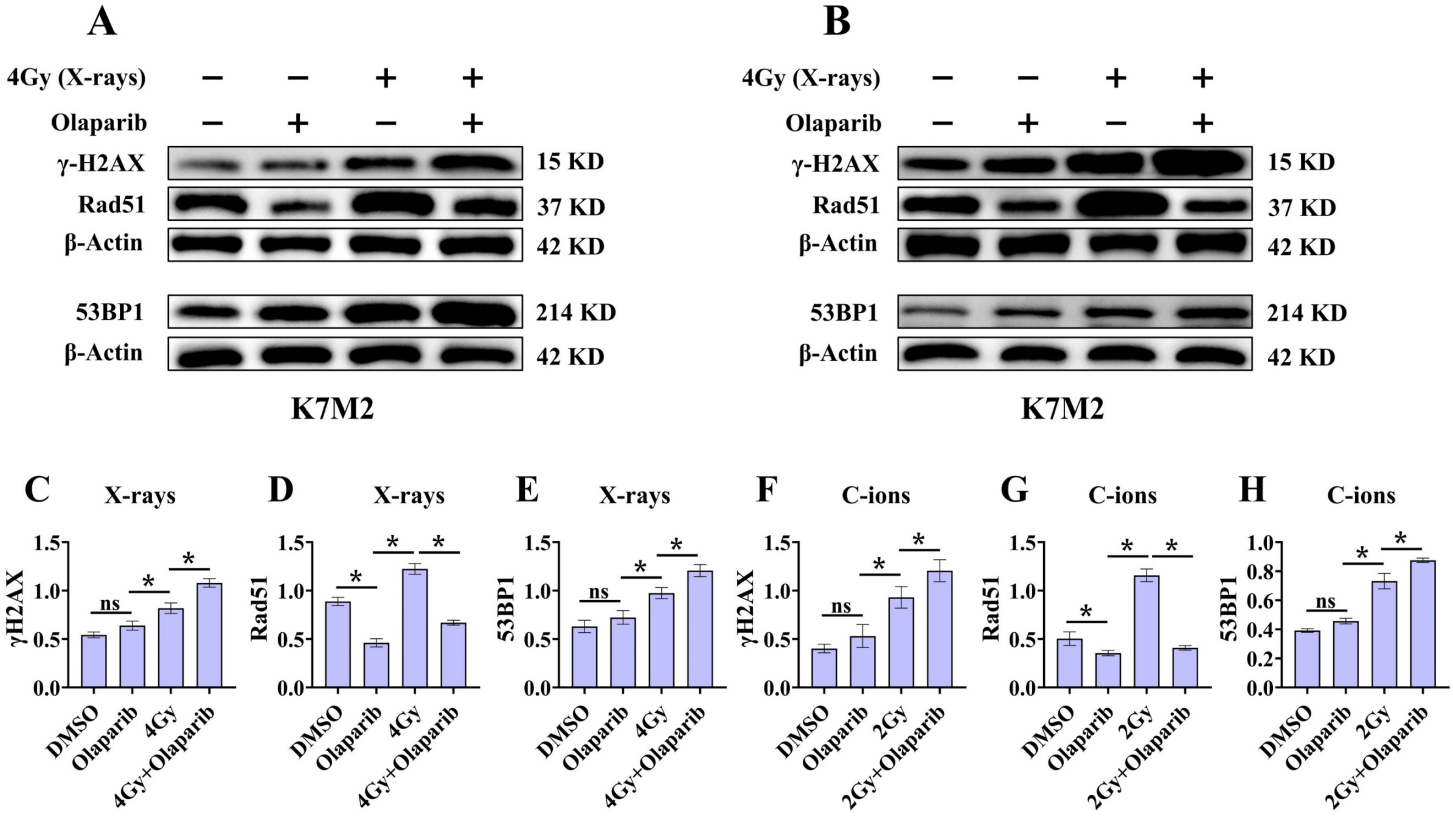
Effect of olaparib on proteins expression of γ‐H2AX, 53BP1, and Rad51 in irradiated K7M2 (A and B) osteosarcoma cell.DMEM medium containing olaparib (10 μM) was added 2 hours before irradiation and western blot assay 12 hours after irradiation. (A) Proteins expression of γ‐H2AX, 53BP1, and Rad51 after X‐rays irradiation for K7M2 cells. (B) Proteins expression of γ‐H2AX, 53BP1, and Rad51 after C‐ions irradiation for K7M2 cells. The relative expression of the proteins (C-H) were shown as mean +/- SD. Data were considered to be significantly different when *p* < 0.05 (*).

Radiation can cause DNA damage through direct and indirect action, resulting in DNA SSBs and DSBs 14, 15. Cell cycle experiment results suggest that osteosarcoma cells displayed significant G_2_/M phase arrest in the olaparib and combination groups compared to that in the control group. This may be because olaparib induces more severe DNA damage. Based on the hypothesis that PARPi may induce DNA damage in irradiated osteosarcoma cells, we monitored the formation of γ‐H2AX foci and 53BP1 foci within 2 hours and 12 hours post-irradiation (Fig. 8A and B; Fig. 9A and B). γ‐H2AX and 53BP1 foci were smaller and circular after exposure to X-rays but were larger and more irregularly shaped after exposure to C-ions (Fig. 8A and B; Fig. 9A and B). Moreover, olaparib induces more γ‐H2AX/53BP1 foci in irradiated osteosarcoma cells (*p* < 0.05). As shown in Fig. 8 and Fig. 9, we observed U2OS and K7M2 foci for 2 hours after irradiation:13.47/12.94 (U2OS_X-rays_) vs. 22.41/15.48 (U2OS_X-rays+olaparib_), 13.49/8.56 (U2OS_C-ions_) vs. 19.14/13.20 (U2OS_C-ions+olaparib_); 14.41/8.41 (K7M2_X-rays_) vs. 23.23/12.68 (K7M2_X-rays+olaparib_), 13.82/9.16 (K7M2_C-ions_) vs. 19.48/14.57 (K7M2_C-ions+olaparib_). Furthermore, olaparib sustained DNA damage in the irradiated osteosarcoma cells (Fig. 8 and 9). 12 hours after irradiation, western blot assay was performed to detect related proteins of the DNA damage (γ‐H2AX and 53BP1) (Fig. 10A and B; Fig. 11A and B). Our results showed that (Fig. 10C, E, F, H; Fig. 11C, E, F, H), compared with the irradiation alone, the combination group induced obvious increase in the expression of γ‐H2AX (U2OS_X-rays_ vs. U2OS_X-rays+olaparib_: 1.64-fold vs. 2.69-fold, *P* < 0.05; U2OS_C-ions_ vs. U2OS_C-ions+olaparib_: 2.07-fold vs. 3.10-fold, *P* < 0.05; K7M2_X-rays_ vs. K7M2_X-rays+olaparib_: 1.51-fold vs. 1.99-fold, *P* < 0.05; K7M2_C-ions_ vs. K7M2_C-ions+olaparib_: 2.31-fold vs. 3.00-fold, *P* < 0.05), and 53BP1 (U2OS_X-rays_ vs. U2OS_X-rays+olaparib_: 1.48-fold vs. 1.79-fold, *P* < 0.05; U2OS_C-ions_ vs. U2OS_C-ions+olaparib_: 1.92-fold vs. 2.86-fold, *P* < 0.05; K7M2_X-rays_ vs. K7M2_X-rays+olaparib_: 1.54-fold vs. 1.91-fold, *P* < 0.05; K7M2_C-ions_ vs. K7M2_C-ions+olaparib_: 1.87-fold vs. 2.23-fold, *P* < 0.05).

### Olaparib inhibits HR repair in irradiated osteosarcoma cells

Because the HR pathway repair is one of the main DBS repair mechanisms, Rad51 foci were indirectly used to quantify the ability of HR repair. We analysed the formation of Rad51 foci in the combination group and found that the number of Rad51 foci per nucleus was significantly lower than that in the irradiated group (*P* < 0.05) (Fig. 8C and D; Fig. 9C and D). As shown in Fig. 8 and Fig. 9, we observed mean Rad51 foci C-ions U2OS and K7M2 for 12 hours after irradiation: 2.67 (U2OS_X-rays_) vs. 1.07 (U2OS_X-rays+olaparib_), 5.08 (U2OS_C-ions_) vs. 2.89 (U2OS_C-ions+olaparib_); 3.64 (K7M2_X-rays_) vs. 2.04 (K7M2_X-rays+olaparib_), 5.64 (K7M2_C-ions_) vs. 3.01 (K7M2_C-ions+olaparib_). 12 hours after irradiation, western blot assay was performed to detect related proteins of the HR repair (Rad51) (Fig. 10A and B; Fig. 11A and B). Our results showed that (Fig. 10D and G; Fig. 11D and G), compared with the irradiation alone, the combination group induced obvious decrease in the expression of Rad51 (U2OS_X-rays_ vs. U2OS_X-rays+olaparib_: 1.51-fold vs. 0.69-fold, *P* < 0.05; U2OS_C-ions_ vs. U2OS_C-ions+olaparib_: 1.84-fold vs. 0.94-fold, *P* < 0.05; K7M2_X-rays_ vs. K7M2_X-rays+olaparib_: 1.38-fold vs. 0.76-fold, *P* < 0.05; K7M2_C-ions_ vs. K7M2_C-ions+olaparib_: 2.30-fold vs. 0.81-fold, *P* < 0.05).

## Discussion

Tumour cells with deficient homologous repair mechanisms are more sensitive to PARPi, including tumours with BRCA1/2 mutations and BRCAness 26, 27. In this study, we analysed the genetic characteristics of U2OS and K7M2 cells via exon sequencing (Supplementary Data 1 and 1). According to DNA repair gene analysis, we observed BRCA1/2 mutations in U2OS cells, especially BRCA2, at a frequency of 99.37% (Table 1), while K7M2 cells showed obvious BRCA2 gene mutation characteristics such as base substitution, frameshift deletion, and frameshift insertion (Table 1). Other common genetic mutations associated with the HR repair pathway were also observed in these two osteosarcoma cell lines (Supplementary Tables 1 and 2): BRIP1, CHEK1, FANCD2, FANCI, FANCM, NBN, and RAD51D (U2OS cells); ATM, ATR, BLM, BRIP1, FANCA, FANCI, FANCL, FANCM, NBN, RAD51C, and RPA1 (K7M2 cells). In addition, XRCC4 mutations associated with the non-homologous end-joining (NHEJ) repair pathway were also observed in K7M2 osteosarcoma cell lines 29. In the olaparib group, 10 µM olaparib inhibited the proliferation of U2OS cells by approximately 20% and 22% cell proliferation activity in K7M2 cells (Fig. 2B and D). About irradiation dose, we selected a dose that reduced the cell proliferation activity of control cells by approximately 50%, corresponding to 4 Gy for X-rays (the cell proliferation activity of U2OS cells was 65.03%±1.6%; the cell proliferation activity of K7M2 cells was 65.74%±3.6%) and 2 Gy for C-ions (the cell proliferation activity of U2OS cells was 42.71%±2.1%; the cell proliferation activity of K7M2 cells was 43.84%±6.81%). Because BRCA1/2 genes and several genes involved in HR repair were mutated in U2OS and K7M2 osteosarcoma cells, co-treatment of olaparib and irradiation may have increased the sensitivity to PARPi due to the "synthetic lethality effect" 26-28, 30. This hypothesis was confirmed by our colony formation, cell migration, cell proliferation, and cell apoptosis assays (Fig. 2, 3, 4, 5 and 6). These results are consistent with those reported by Jannetti et al. 31.

After the cells were irradiated, DNA damage checkpoints were activated, which delayed the cell cycle and provided time for the repair of radiation damage 32, 33. Cell cycle progression may show different changes in response to different LET irradiations. In general, a high LET may induce more pronounced G_2_/M arrest 34. In our study, we observed that high-LET C-ions induced more severe G_2_/M arrest in U2OS and K7M2 osteosarcoma cells than did low-LET X-rays (Fig. 7). Meanwhile, the proportion of cells in the G_2_/M phase in the combination group was remarkably higher than that in the irradiation group, suggesting that olaparib combined with irradiation can further induce cell cycle redistribution (increased the number of cells in G_2_/M phase), which increased the radiosensitization of osteosarcoma cells to X-rays and C-ions (Fig. 7). This arousal strategy is one way to improve radiosensitization of radiation-resistant tumours 35.

Radiation can cause DNA damage through direct and indirect action, resulting in DNA SSBs and DSBs 14, 15. Cell cycle experiment results suggest that osteosarcoma cells displayed significant G_2_/M phase arrest in the olaparib and combination groups compared to that in the control group. This may be because olaparib induces more severe DNA damage. Based on the hypothesis that PARPi may induce DNA damage in irradiated osteosarcoma cells, we monitored the formation of γ‐H2AX foci and 53BP1 foci post-irradiation (Fig. 8A and B; Fig. 9A and B). Larger and more irregularly shaped γ‐H2AX foci are critical for RT and can induce more severe clustered DNA damage. Generally, it is difficult to repair such clustered DNA damage 36, 37. In this study, our results revealed that olaparib induced more severe clustered DNA damage in irradiated osteosarcoma cells, rendering γ‐H2AX/53BP1 foci to be larger and more irregularly shaped relative to irradiation alone (Fig. 8 and Fig. 9). After 12 hours of irradiation, many γ‐H2AX/53BP1 foci were retained in the combined group than in the irradiation group, suggesting that olaparib delayed DNA damage repair and reduced kinetics in irradiated osteosarcoma cells (Fig. 8 and Fig. 9). The results of western blot assay were consistent with immunofluorescence, the γ‐H2AX/53BP1 protein expression in the combination group was higher than that in the irradiated group (Fig. 10 and Fig 11). This may be attributed to the presence of BRCA1/2 gene mutations and other genetic mutations associated with the HR repair pathway in the U2OS and K7M2 osteosarcoma cell lines (Supplementary Tables 1 and 2), which causes homologous recombination deficiency (HRD) 38. Moreover, HRD led to genomic instability and accumulation of DNA damage in osteosarcoma cells after irradiation (Fig. 8 and Fig. 9). Overall, these results suggest that olaparib improves the radiation sensitivity of osteosarcoma cells by inducing complex DNA damage.

Previous studies have shown that mutations in the BRCA1/2 gene lead to an inability to synthesise an active protein that deals with DNA end damage, resulting in inefficient "repair" and accumulation of DNA damage 26, 27. Normally, the BRCA2 protein binds to the Rad51 protein, preventing Rad51 from polymerising with the DNA and leaving it in its inactive form 39, 40. In the event of DNA damage, BRCA2 helps to quickly carry the Rad51 protein to the site of the damage to perform HR repair 39, 40. In the present immunofluorescence assay and western blot assay, the amount of Rad51 foci and Rad51 protein expression in the combination group was lower than that in the irradiated group (Fig. 8C and D; Fig. 9C and D; Fig. 10A and B; Fig. 11A and B), suggesting that olaparib may inhibit HR repair by regulating Rad51 recruitment at sites of DNA damage in response to radiation. Based on the above results, it can be further speculated that the radiosensitization effect of olaparib on U2OS and K7M2 osteosarcoma cells may be mediated by the inhibition of HR repair.

## Conclusion

Our results revealed that the PARP inhibitor olaparib combined with irradiation (X-rays or C-ions) enhanced the radiosensitivity of osteosarcoma cell lines (U2OS and K7M2). Radiosensitization mechanisms for osteosarcoma cells through this combination strategy include the inhibition of proliferation, inhibition of cell migration, induction of apoptosis, G_2_/M arrest, accumulated complex DNA damage, and inhibition of DNA damage repair. Genetic tests are important prerequisites for evaluating the potential of olaparib in radiosensitization. Finally, our findings provide a potential theoretical basis for the clinical application of olaparib in overcoming radiation resistance in osteosarcoma.

## Supplementary Material

Supplementary Data 1 Analysis of specific mutations in U2OS cells.

Supplementary Data 2 Analysis of specific mutations in K7M2 cells.

Supplementary Table 1 Analysis of specific nonsynonymous mutations in U2OS cells.

Supplementary Table 2 Analysis of specific nonsynonymous mutations in K7M2 cells.

## Figures and Tables

**Table 1:** Analysis of BRCA1 and BRCA2 mutations in U2OS and K7M2 cells

Gene	Transcript^a^	Exons	Description	Expected consequence^b^	Mutated allele frequency	Interpretation
**U2OS cell**						
***BRCA1***	NM_007297	Ex14 Ex15 Ex16	c.4696T>C	p.S1566G	34.96%	Nonsynonymous
	NM_007297	Ex9 Ex10	c.3407T>C	p.K1136R	34.90%	Nonsynonymous
	NM_007297	Ex9 Ex10	c.2972T>C	p.E991G	34.29%	Nonsynonymous
	NM_007297	Ex9 Ex10	c.2471G>A	p.P824L	41.00%	Nonsynonymous
***BRCA2***	NM_000059	Ex14	c.7397T>C	p.V2466A	99.37%	Nonsynonymous
						
**K7M2 cell**						
***BRCA2***	NM_001081001	Ex11	c.4524_4525del	p.I1508fs	100%	Frameshift deletion
	NM_001081001	Ex11	c.4528_4529insAA	p.Q1510fs	100%	Frameshift insertion
	NM_001081001	Ex11	c.5695_5696insTTT	p.A1899delinsVS	25.00%	Nonframeshift insertion
	NM_001081001	Ex11	c.5708_5709insTTGc.	p.P1903delinsPCD	20.00%	Nonframeshift insertion
	NM_001081001	Ex11	5698G>A	p.A1900T	25.00%	Nonsynonymous
	NM_001081001	Ex11	c.5699C>A	p.A1900E	25.00%	Nonsynonymous
	NM_001081001	Ex11	c.5702C>T	p.T1901I	25.00%	Nonsynonymous
	NM_001081001	Ex11	c.5704C>T	p.P1902S	25.00%	Nonsynonymous
	NM_001081001	Ex11	c.5713G>A	p.G1905S	18.18%	Nonsynonymous
	NM_001081001	Ex11	c.5726G>C	p.W1909S	16.67%	Nonsynonymous
	NM_001081001	Ex11	c.5727G>A	p.W1909X	16.67%	Nonsynonymous
	NM_001081001	Ex11	c.5732C>T	p.T1911I	15.38%	Nonsynonymous
	NM_001081001	Ex11	c.5734A>T	p.S1912C	15.38%	Nonsynonymous
	NM_001081001	Ex18	c.7988G>A	p.S2663N	12.50%	Nonsynonymous
	NM_001081001	Ex18	c.8020A>C	p.T2674P	13.04%	Nonsynonymous
	NM_001081001	Ex18	c.8021C>T	p.T2674I	13.04%	Nonsynonymous
	NM_001081001	Ex18	c.8025G>T	p.Q2675H	13.04%	Nonsynonymous
	NM_001081001	Ex25	c.9175G>T	p.G3059C	100%	Nonsynonymous

^a^ Mutation nomenclature per HGVS recommendations. ^b^ Expected consequence on protein level.

**Table 2:** Calculated parameters of U2OS and K7M2 cell survival after irradiation with X-rays and C-ions with and without olaparib (from Fig. 2)

	D10 ^a^	D37 ^b^	ER (D10) ^c^	ER (D37) ^d^	ER ^e^
U2OS					
X-rays	5.48	3.38	/	/	
X-rays+ olaparib	4.49	2.10	1.22	1.61	1.42
C-ions	1.91	1.06	/	/	
C-ions+ olaparib	1.40	0.61	1.36	1.74	1.55
K7M2					
X-rays	3.42	2.00	/	/	
X-rays+olaparib	2.36	1.11	1.45	1.80	1.63
C-ions	1.95	1.08	/	/	
C-ions+ olaparib	1.24	0.57	1.57	1.89	1.73

^a^ the D10 dose gives a surviving fraction of 0.1. ^b^ the D37 dose gives a surviving fraction of 0.37. ^c^ ER (D10) values are calculated as: D10 (with olaparib) / D10 (without olaparib) for each irradiation quality. ^d^ ER (D37) values are calculated as: D37 (with olaparib) / D37 (without olaparib) for each irradiation quality. ^e^ 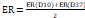

## Abbreviations

RT: radiotherapy
C-ions RT: carbon ions radiotherapy
RBE: relative biological effectiveness
LC: local control
SSBs: single strand breaks
BER: base excision repair
DSBs: double strand breaks
PARP: Poly (ADP-ribose) polymerases
PARPi: PARP inhibitors
HR: homologous recombination
FBS: fetal bovine serum
HIMM: Heavy Ion Medical Machine
LET: linear energy transfer
GSEA: Gene Set Enrichment Analysis
CCK-8: cell count kit-8
OD: optical density
PE: plating efficiency
SF: survival fraction
EDTA: ethylene diamine tetraacetic acid
PI: propidium iodide
BSA: bovine serum albumin
ER: enhancement ratio
PMSF: phenylmethanesulfonyl fluoride
SDS-PAGE: sodium dodecyl sulfate-polyacrylamide gel electrophoresis
NHEJ: non-homologous end-joining
HRD: homologous recombination deficiency

## References

[1] Esiashvili N, Goodman M, Marcus RB Jr (2008). Changes in incidence and survival of Ewing sarcoma patients over the past 3 decades: Surveillance Epidemiology and End Results data. J Pediatr Hematol Oncol.

[2] Mirabello L, Troisi RJ, Savage SA (2009). Osteosarcoma incidence and survival rates from 1973 to 2004: data from the Surveillance, Epidemiology, and End Results Program. Cancer.

[3] Liu S, Cai X, Qiu L (2020). Interpretation of the new WHO classification of bone tumors. Chin J Magn Reson Imaging.

[4] Eaton BR, Schwarz R, Vatner R (2021). Osteosarcoma. Pediatr Blood Cancer.

[5] Kawaguchi N, Ahmed AR, Matsumoto S, Manabe J, Matsushita Y (2004). The concept of curative margin in surgery for bone and soft tissue sarcoma. Clin Orthop Relat Res.

[6] Tinkle CL, Lu J, Han Y (2019). Curative-intent radiotherapy for pediatric osteosarcoma: The St. Jude experience. Pediatr Blood Cancer.

[7] Dangoor A, Seddon B, Gerrand C, Grimer R, Whelan J, Judson I (2016). UK guidelines for the management of soft tissue sarcomas. Clin Sarcoma Res.

[8] Schwarz R, Bruland O, Cassoni A, Schomberg P, Bielack S (2009). The role of radiotherapy in oseosarcoma. Cancer Treat Res.

[9] Ciernik IF, Niemierko A, Harmon DC (2011). Proton-based radiotherapy for unresectable or incompletely resected osteosarcoma. Cancer.

[10] Dong M, Liu R, Zhang Q (2022). Efficacy and safety of carbon ion radiotherapy for bone sarcomas: a systematic review and meta-analysis. Radiat Oncol.

[11] Kamada T, Tsujii H, Blakely EA (2015). Carbon ion radiotherapy in Japan: an assessment of 20 years of clinical experience. Lancet Oncol.

[12] Hall N Hadron therapy: how nuclear research is improving human health for Medicine. NUPECC: Nuclear Phys. (2014). http://www.nupecc.org/pub/npmed2014_brochure.pdf.

[13] Matsunobu A, Imai R, Kamada T (2012). Impact of carbon ion radiotherapy for unresectable osteosarcoma of the trunk. Cancer.

[14] Murai J (2017). Targeting DNA repair and replication stress in the treatment of ovarian cancer. Int J Clin Oncol.

[15] Thomas A, Murai J, Pommier Y (2018). The evolving landscape of predictive biomarkers of response to PARP inhibitors. J Clin Invest.

[16] Morales J, Li L, Fattah FJ (2014). Review of poly (ADP-ribose) polymerase (PARP) mechanisms of action and rationale for targeting in cancer and other diseases. Crit Rev Eukaryot Gene Expr.

[17] Nickoloff JA, Jones D, Lee SH, Williamson EA, Hromas R (2017). Drugging the Cancers Addicted to DNA Repair. J Natl Cancer Inst.

[18] Annunziata CM, O'Shaughnessy J (2010). Poly (ADP-ribose) polymerase as a novel therapeutic target in cancer. Clin Cancer Res.

[19] Ray Chaudhuri A, Nussenzweig A (2017). The multifaceted roles of PARP1 in DNA repair and chromatin remodelling. Nat Rev Mol Cell Biol.

[20] Jonuscheit S, Jost T, Gajdošová F (2021). PARP Inhibitors Talazoparib and Niraparib Sensitize Melanoma Cells to Ionizing Radiation. Genes (Basel).

[21] Lakomy DS, Urbauer DL, Westin SN, Lin LL (2019). Phase I study of the PARP inhibitor talazoparib with radiation therapy for locally recurrent gynecologic cancers. Clin Transl Radiat Oncol.

[22] Césaire M, Ghosh U, Austry JB (2019). Sensitization of chondrosarcoma cells with PARP inhibitor and high-LET radiation. J Bone Oncol.

[23] Lesueur P, Chevalier F, El-Habr EA (2018). Radiosensitization Effect of Talazoparib, a Parp Inhibitor, on Glioblastoma Stem Cells Exposed to Low and High Linear Energy Transfer Radiation. Sci Rep.

[24] Bi Y, Verginadis II, Dey S (2018). Radiosensitization by the PARP inhibitor olaparib in BRCA1-proficient and deficient high-grade serous ovarian carcinomas. Gynecol Oncol.

[25] Liu C, Gross N, Li Y (2020). PARP inhibitor Olaparib increases the sensitization to radiotherapy in FaDu cells. J Cell Mol Med.

[26] Ashworth A, Lord CJ (2018). Synthetic lethal therapies for cancer: what's next after PARP inhibitors?. Nat Rev Clin Oncol.

[27] Lee JM, Ledermann JA, Kohn EC (2014). PARP Inhibitors for BRCA1/2 mutation-associated and BRCA-like malignancies. Ann Oncol.

[28] Kovac M, Blattmann C, Ribi S (2015). Exome sequencing of osteosarcoma reveals mutation signatures reminiscent of BRCA deficiency. Nat Commun.

[29] Chen S, Lee L, Naila T (2021). Structural basis of long-range to short-range synaptic transition in NHEJ. Nature.

[30] Farmer H, McCabe N, Lord CJ (2005). Targeting the DNA repair defect in BRCA mutant cells as a therapeutic strategy. Nature.

[31] Jannetti SA, Zeglis BM, Zalutsky MR, Reiner T (2020). Poly(ADP-Ribose)Polymerase (PARP) Inhibitors and Radiation Therapy. Front Pharmacol.

[32] Ferguson AM, White LS, Donovan PJ, Piwnica-Worms H (2005). Normal cell cycle and checkpoint responses in mice and cells lacking Cdc25B and Cdc25C protein phosphatases. Mol Cell Biol.

[33] Kawano M, Umeda S, Yasuda T (2016). FGF18 signaling in the hair cycle resting phase determines radioresistance of hair follicles by arresting hair cycling. Adv Radiat Oncol.

[34] Zhang J, Si J, Gan L, Zhou R, Guo M, Zhang H (2021). Harnessing the targeting potential of differential radiobiological effects of photon versus particle radiation for cancer treatment. J Cell Physiol.

[35] Recasens A, Munoz L (2019). Targeting Cancer Cell Dormancy. Trends Pharmacol Sci.

[36] Girdhani S, Sachs R, Hlatky L (2013). Biological effects of proton radiation: what we know and don't know. Radiat Res.

[37] Lopez Perez R, Nicolay NH, Wolf JC (2019). DNA damage response of clinical carbon ion versus photon radiation in human glioblastoma cells. Radiother Oncol.

[38] Pellegrino B, Mateo J, Serra V, Balmaña J (2019). Controversies in oncology: are genomic tests quantifying homologous recombination repair deficiency (HRD) useful for treatment decision making?. ESMO Open.

[39] Richardson C (2005). RAD51, genomic stability, and tumorigenesis. Cancer Lett.

[40] Moynahan ME, Jasin M (2010). Mitotic homologous recombination maintains genomic stability and suppresses tumorigenesis. Nat Rev Mol Cell Biol.

