# Supplementary material for: Radiosensitization of Osteosarcoma Cells Using the PARP Inhibitor Olaparib Combined with X-rays or Carbon Ions
Source: J Cancer. 2024 Jan 1;15(3):699–713. doi: 10.7150/jca.90371 (PMC10777037; doi:10.7150/jca.90371)
Supplement: Supplementary file 3 — Supplementary Table 1 Analysis of specific nonsynonymous mutations in U2OS cells. [file jcav15p0699s3.pdf]

Supplementary Table 1 Analysis of specific nonsynonymous mutations in U2OS cells

Gene	Transcript[a]	Exons	Description	Expected consequence[b]	Mutated allele frequency	Function
AURKA	NM_003600	Ex3 Ex4 Ex5	c.169T>C	p.I57V	84.82%	Nonsynonymous
BARD1	NM_001282543	Ex5 Ex6	c.1462C>T	p.V488M	37.83%	Nonsynonymous
	NM_001282543	Ex3 Ex4	c.1077C>G	p.R359S	54.87%	Nonsynonymous
	NM_000465	Ex1	c.70G>A	p.P24S	43.77%	Nonsynonymous
BRCA1	NM_007297	Ex14 Ex15 Ex16	c.4696T>C	p.S1566G	34.96%	Nonsynonymous
	NM_007297	Ex9 Ex10	c.3407T>C	p.K1136R	34.90%	Nonsynonymous
	NM_007297	Ex9 Ex10	c.2972T>C	p.E991G	34.29%	Nonsynonymous
	NM_007297	Ex9 Ex10	c.2471G>A	p.P824L	41.00%	Nonsynonymous
BRCA2	NM_000059	Ex14	c.7397T>C	p.V2466A	99.37%	Nonsynonymous
BRIP1	NM_032043	Ex19	c.2755A>G	p.S919P	59.72%	Nonsynonymous
CHEK1	NM_001244846	Ex12 Ex13	c.1309A>G	p.I437V	97.26%	Nonsynonymous
ERCC2	NM_000400	Ex23	c.2251T>G	p.K751Q	33.08%	Nonsynonymous
	NM_001130867	Ex9 Ex10	c.862C>T	p.D288N	37.88%	Nonsynonymous
FANCD2	NM_001018115	Ex15	c.1214A>G	p.N405S	42.01%	Nonsynonymous
FANCI	NM_001113378	Ex4	c.257C>T	p.A86V	35.69%	Nonsynonymous
FANCM	NM_001308133	Ex13 Ex14	c.2554G>T	p.V852L	15.68%	Nonsynonymous
	NM_001308133	Ex15 Ex16	c.4300A>G	p.I1434V	12.00%	Nonsynonymous
	NM_001308133	Ex20 Ex21	c.5356C>G	p.P1786A	11.58%	Nonsynonymous
NBN	NM_002485	Ex5 Ex6	c.553C>G	p.E185Q	34.53%	Nonsynonymous
PIK3R2	NM_005027	Ex6	c.700A>C	p.S234R	89.61%	Nonsynonymous
	NM_005027	Ex8	c.937T>C	p.S313P	95.01%	Nonsynonymous
RAD51D	NM_133629	Ex3 Ex6	c.158C>T	p.R53Q	15.93%	Nonsynonymous
RNF168	NM_152617	Ex6	c.1202G>T	p.P401Q	54.05%	Nonsynonymous
SMARCA2	NM_001289398	Ex6 Ex7 Ex32 Ex33	c.612C>G	p.D204E	16.82%	Nonsynonymous
TDG	NM_003211	Ex3	c.287dupA	p.E96fs	44.89%	Frameshift insertion
	NM_001363612	Ex9	c.670G>A	p.V224M	10.81%	Nonsynonymous
TOPBP1	NM_001363889	Ex14	c.2435G>A	p.S812L	14.11%	Nonsynonymous
	NM_001363889	Ex10	c.1354T>G	p.K452Q	71.59%	Nonsynonymous
UIMC1	NM_001199298	Ex8 Ex9	c.1304G>A	p.P435L	14.25%	Nonsynonymous
XRCC3	NM_001100118	Ex7 Ex8	c.722G>A	p.T241M	30.75%	Nonsynonymous

[a] Mutation nomenclature per HGVS recommendations.

[b] Expected consequence on protein level.
