# Supplementary material for: Radiosensitization of Osteosarcoma Cells Using the PARP Inhibitor Olaparib Combined with X-rays or Carbon Ions
Source: J Cancer. 2024 Jan 1;15(3):699–713. doi: 10.7150/jca.90371 (PMC10777037; doi:10.7150/jca.90371)
Supplement: Supplementary file 4 — Supplementary Table 2 Analysis of specific nonsynonymous mutations in K7M2 cells. [file jcav15p0699s4.pdf]

Supplementary Table 2 Analysis of specific nonsynonymous mutations in K7M2 cells

Gene	Transcript[a]	Exons	Description	Expected consequence[b]	Mutated allele frequency	Function
ARID1A	NM_001080819	Ex1	c.783_785del	p.261_262del	100%	Nonframeshift deletion
	NM_001080819	Ex20	c.5396T>C	p.N1799S	100%	Nonsynonymous
	NM_001080819	Ex20	c.5264T>G	p.Y1755S	100%	Nonsynonymous
ATM	NM_007499	Ex25	c.3642_3643del	p.I1214fs	100%	Frameshift deletion
	NM_007499	Ex25	c.3639_3640insGC	p.I1214fs	100%	Frameshift insertion
ATR	NM_019864	Ex4	c.904C>G	p.L302V	92.59%	Nonsynonymous
	NM_019864	Ex32	c.5447C>T	p.T1816I	22.22%	Nonsynonymous
	NM_019864	Ex32	c.5452A>G	p.T1818A	22.22%	Nonsynonymous
	NM_019864	Ex32	c.5464A>T	p.T1822S	22.22%	Nonsynonymous
ATRX	NM_009530	Ex25	c.5857C>T	p.V1953M	100%	Nonsynonymous
BABAM1	NM_026636	Ex2	c.67A>C	p.T23P	100%	Nonsynonymous
	NM_026636	Ex2	c.143G>A	p.S48N	100%	Nonsynonymous
	NM_026636	Ex9	c.943G>T	p.A315S	100%	Nonsynonymous
BLM	NM_001042527	Ex7	c.1719_1721del	p.573_574del	100%	Nonframeshift deletion
	NM_001042527	Ex20	c.3883C>A	p.V1295L	100%	Nonsynonymous
	NM_001042527	Ex7	c.1862G>T	p.T621N	100%	Nonsynonymous
	NM_001042527	Ex7	c.1771T>C	p.T591A	100%	Nonsynonymous
	NM_001042527	Ex7	c.1640T>G	p.N547T	100%	Nonsynonymous
	NM_001042527	Ex7	c.1636A>G	p.W546R	100%	Nonsynonymous
	NM_001042527	Ex7	c.1603C>T	p.V535M	100%	Nonsynonymous
	NM_001042527	Ex3	c.392A>G	p.L131P	100%	Nonsynonymous
BRCA2	NM_001081001	Ex11	c.4524_4525del	p.I1508fs	100%	Frameshift deletion
	NM_001081001	Ex11	c.4528_4529insAA	p.Q1510fs	100%	Frameshift insertion
	NM_001081001	Ex11	c.5695_5696insTTT	p.A1899delinsVS	25.00%	Nonframeshift insertion
	NM_001081001	Ex11	c.5708_5709insTTG	p.P1903delinsPCD	20.00%	Nonframeshift insertion
	NM_001081001	Ex11	c.5698G>A	p.A1900T	25.00%	Nonsynonymous
	NM_001081001	Ex11	c.5699C>A	p.A1900E	25.00%	Nonsynonymous
	NM_001081001	Ex11	c.5702C>T	p.T1901I	25.00%	Nonsynonymous
	NM_001081001	Ex11	c.5704C>T	p.P1902S	25.00%	Nonsynonymous
	NM_001081001	Ex11	c.5713G>A	p.G1905S	18.18%	Nonsynonymous
	NM_001081001	Ex11	c.5726G>C	p.W1909S	16.67%	Nonsynonymous
	NM_001081001	Ex11	c.5727G>A	p.W1909X	16.67%	Nonsynonymous
	NM_001081001	Ex11	c.5732C>T	p.T1911I	15.38%	Nonsynonymous
	NM_001081001	Ex11	c.5734A>T	p.S1912C	15.38%	Nonsynonymous
	NM_001081001	Ex18	c.7988G>A	p.S2663N	12.50%	Nonsynonymous
	NM_001081001	Ex18	c.8020A>C	p.T2674P	13.04%	Nonsynonymous
	NM_001081001	Ex18	c.8021C>T	p.T2674I	13.04%	Nonsynonymous
	NM_001081001	Ex18	c.8025G>T	p.Q2675H	13.04%	Nonsynonymous
	NM_001081001	Ex25	c.9175G>T	p.G3059C	100%	Nonsynonymous
BRIP1	NM_178309	Ex10	c.1445>T	p.R482K	100%	Nonsynonymous
	NM_178309	Ex10	c.1441A>G	p.Y481H	100%	Nonsynonymous
	NM_178309	Ex10	c.1436T>G	p.N479T	100%	Nonsynonymous
ERCC1	NM_001127324	Ex3	c.163G>A	p.V55M	100%	Nonsynonymous
FANCA	NM_016925	Ex37	c.3616C>A	p.A1206S	100%	Nonsynonymous
	NM_016925	Ex33	c.3280T>C	p.I1094V	100%	Nonsynonymous
	NM_016925	Ex33	c.3271C>T	p.G1091R	100%	Nonsynonymous
	NM_016925	Ex31	c.3014C>T	p.R1005H	100%	Nonsynonymous
	NM_016925	Ex29	c.2828A>G	p.M943T	100%	Nonsynonymous
	NM_016925	Ex28	c.2695G>T	p.R899S	100%	Nonsynonymous
	NM_016925	Ex28	c.2624G>A	p.A875V	100%	Nonsynonymous
	NM_016925	Ex20	c.1797C>A	p.L599F	100%	Nonsynonymous
	NM_016925	Ex5	c.439T>C	p.M147V	100%	Nonsynonymous
	NM_016925	Ex4	c.356C>T	p.R119H	100%	Nonsynonymous
FANCG	NM_001163233	Ex10	c.1338G>C	p.C446W	22.73%	Nonsynonymous
FANCI	NM_145946	Ex24	c.2533A>G	p.T845A	98.31%	Nonsynonymous
FANCL	NM_001277273	Ex1	c.5A>C	p.D2A	100%	Nonsynonymous
FANCM	NM_001364447	Ex23	c.3736A>C	p.I1246L	100%	Nonsynonymous
	NM_001364447	Ex23	c.3791A>T	p.Q1264L	100%	Nonsynonymous
NBN	NM_013752	Ex5	c.514A>G	p.I172V	12.90%	Nonsynonymous
	NM_013752	Ex5	c.532T>A	p.S178T	12.12%	Nonsynonymous
	NM_013752	Ex9	c.1098C>G	p.D366E	100%	Nonsynonymous
PIK3R2	NM_008841	Ex11	c.1297T>C	p.I433V	100%	Nonsynonymous
RAD51C	NM_053269	wholegene	/	/	71.43%	Frameshift deletion
RPA1	NM_026653	Ex6	c.399_404del	p.133_135del	94.66%	Nonframeshift deletion
	NM_001164223	Ex7	c.462_467del	p.154_156del	94.66%	Nonframeshift deletion
UIMC1	NM_001293660	Ex6 Ex8	c.506G>A	p.T169I	100%	Nonsynonymous
XRCC4	NM_028012	Ex4	c.375A>T	p.D125E	99.51%	Nonsynonymous
	NM_028012	Ex3	c.278C>T	p.R93Q	100%	Nonsynonymous

[a] Mutation nomenclature per HGVS recommendations.

[b] Expected consequence on protein level.
